# Supplementary material for: A Unique Gene Regulatory Network Resets the Human Germline Epigenome for Development
Source: Cell. 2015 Jun 4;161(6):1453–67. doi: 10.1016/j.cell.2015.04.053 (PMC4459712; doi:10.1016/j.cell.2015.04.053)
Supplement: Document S1. Supplemental Experimental Procedures and Table S4 [file mmc1.pdf]

Cell, Volume *161*

Supplemental Information

A Unique Gene Regulatory Network Resets the Human Germline Epigenome for Development

Walfred W.C. Tang, Sabine Dietmann, Naoko Irie, Harry G. Leitch, Vasileios I. Floros, Charles R. Bradshaw, Jamie A. Hackett, Patrick F. Chinnery, and M. Azim Surani

SUPPLEMENTAL EXPERIMENTAL PROCEDURES

Isolation of hPGCs by FACS

Human embryonic genital ridges from individual embryo (Wk5.5, Wk7 and Wk9) were dissected in PBS and separated from surrounding mesonephric tissues. The embryonic tissues were dissociated with 100 μl TrypLE Express (Life Technologies) at 37°C for 20-40 minutes (depending on the size of the tissue). Tissues were pipette up and down for ten times every 5 minutes to facilitate dissociation into single cell suspension. After that, samples were diluted with 100 μl FACS medium (PBS with 3% fetal calf serum & 5 mM EDTA) and centrifuged at 500 xg for 5 minutes. Cell pellet was suspended with FACS medium and incubated with 5 μl of Alexa Fluor 488-conjugated anti-alkaline phosphatase (BD Pharmingen, 561495) and 25 μl of PerCP-Cy5.5-conjugated anti-CD117 (BD Pharmingen 333950) antibodies for 15 minutes at room temperature with rotation at 10 revolutions per minutes (rpm) in dark. Cell suspension was then diluted in 1ml FACS medium and centrifuged at 500 xg for 5 minutes. After removing the supernatant, the cell pellet was resuspended in FACS medium and passed through a 35μm cell strainer. FACS was performed with S3 Cell Sorter (Bio-Rad) and analyses were performed by FlowJo software. Various cell populations were sorted onto Poly-L-Lysine Slides (Thermo Scientific) and fixed in 4% PFA. Alkaline phosphatase staining was performed with Leukocyte Alkaline Phosphatase Kit (Sigma) to determine the purity of hPGCs. Only samples with >97% purity were used for RNA-Seq and BS-Seq.

Conventional ESC Culture

Conventional H9 ESCs were maintained on irradiated MEFs (GlobalStem) in DMEM/F12+GlutaMAX supplemented with 20% KSR, 0.1 mM nonessential amino acids, 0.1

mM 2-mercaptoethanol (all Gibco) and 10-20 ng/ml of bFGF (SCI). Media were replaced everyday. Cells were passaged every 4-6 days using 1 mg/ml of Dispase (Gibco). ROCK inhibitor (Y-27632, TOCRIS bioscience) (10 μM) was supplemented to culture media for 24 hours after passage. For RNA-Seq and BS-Seq, SSEA4-positive conventional H9 ESCs were obtained by FACS with PE-conjugated anti-SSEA4 antibody (BD Pharmingen, 561128).

hPGCLC induction

hPGCLC induction was performed as previously described (Irie et al., 2015). Briefly, NANOS3-mCherry WIS2 ESCs were grown on irradiated mouse embryonic fibroblasts (MEFs) (GlobalStem) in "4i" medium consisting of knockout DMEM supplemented with 20% knockout serum replacement (KSR), 2 mM L-glutamine, 0.1 mM nonessential amino acids, 0.1 mM 2-mercaptoethanol (all Gibco), 20 ng/ml human LIF (Stem Cell Institute (SCI), Cambridge, UK), 8 ng/ml bFGF (SCI), 1 ng/ml TGF-β1 (Peprotech), 3 μM CHIR99021 (Miltenyi Biotec), 1 μM PD0325901 (Miltenyi Biotec), 5 μM SB203580 (TOCRIS bioscience) and 5 μM SP600125 (TOCRIS bioscience). 4i ESCs were dissociated with TrypLE Express and filtered with 50 μm cell filter (PERTEC) and plated to ultra-low cell attachment U-bottom 96-well plates (Corning Costar, 7007), at a density of 4000 cells/well in 80 μl PGCLC medium. PGCLC medium was composed of Glasgow's MEM (GMEM, GIBCO), 15% KSR, 0.1 mM nonessential amino acids, 0.1 mM 2-mercaptoethanol, 100 U/ml Penicillin-0.1 mg/ml Streptomycin, 2 mM L-Glutamine, 1 mM Sodium pyruvate and the following cytokines: 500 ng/ml BMP4 (R&D Systems) or BMP2 (SCI), 1 μg/ml human LIF (SCI), 100 ng/ml SCF (R&D Systems), 50 ng/ml EGF (R&D Systems) and 10 μM ROCK inhibitor. Day 4 and 5 NANOS3-mCherry and CD38-double positive hPGCLCs and surrounding soma (double negative) were collected for 5mC and 5hmC analyses.

BLIMP1 mutant NANOS3-mCherry WIS2 ESCs were generated previously by CRISPR as described (Irie et al., 2015). The mutant clone used in this study harbored a “G” insertion and a “GGTCG” deletion at the two alleles of *BLIMP1* locus in exon 5 respectively. This resulted in frame-shifted transcripts with the absence of BLIMP1 protein as determined by immunofluorescence. hPGCLC induction was performed with *BLIMP1* mutant 4i ESCs as above, but included 2 days pre-induction treatment in N2B27/bFGF/TGF-β1 medium, for direct comparison with published wild-type hPGCLCs RNA-Seq data (Irie et al., 2015). TNAP-single positive *BLIMP1* mutant hPGCLCs were collected at day 4 and subjected to RNA-Seq.

Detection of 5mC and 5hmC at ICR and promoters

4i ESCs, day 4 and day 5 hPGCLCs DNA were extracted by DNeasy Blood and Tissue Kit (Qiagen). Quantitative measurements of 5mC and 5hmC were determined using a modified protocol from the Quest 5hmC Detection Kit (Zymo Research) as previously described (Hackett et al., 2013). Briefly, we introduced an additional *HpaII* digestion to determine 5mC levels at specific CCGG genomic sites. *MspI* digestion was inhibited by glucosylated 5hmC allowing quantification of 5hmC. *HpaII* was inhibited by both 5mC and 5hmC. Therefore subtraction of the *MspI* digest quantification from *HpaII* digest quantification indicates the level of 5mC. The 0% baseline for the assay was set by digesting unglucosylated DNA with *MspI*, while the 100% threshold was set by unmodified and undigested DNA, with the percentage levels of other reactions determined by regression from these parameters. Four reactions were set up for each sample (glucosylated+*MspI*; unglucosylated+*HpaII*; unglucosylated+*MspI*; unglucosylated undigested) each containing buffer and equal amounts of DNA (~20 ng), and incubated according to the manufacturer’s instructions. Subsequently samples were heat inactivated (10 min at 80˚C) and digestion resistant DNA was quantified by quantitative-PCR (qPCR) in

technical quadruplicate using QuantStudio 6 Flex Real-Time PCR System (Applied Biosystems) and KAPA SYBR Fast qPCR mix (KAPA Biosystems). Specific primers that spanned a single CCGG site of interest within an imprinted control region (ICR) or gene promoter were used for amplification with annealing/extension at 62.5˚C for 45 seconds (Table S4). DNA loading was controlled for by normalising to a primer set that spans a region lacking a *HpaII/MspI* restriction site (Chr12 Control F/R).

Immunofluorescence

Human embryonic tissues were fixed in 4% paraformaldehyde (PFA) for 2 hours at 4°C. Fixed tissues were prepared as 8 μm cryosections and immunofluorescence was performed as previously described (Irie et al., 2015). For immunofluorescence of 5mC, 5hmC, H3K27me3 and H3K9me3, slides were subjected to heat-induced epitope retrieval in TE buffer (pH8) at around 95°C by a microwave oven for 40 minutes before permeabilization and primary antibody incubations. Confocal imaging was performed with Leica TCS SP5/SP8 or Zeiss LSM 510 microscopes.

Primary antibodies used were: Rabbit anti-5hmC (1:500, Active Motif, 39769), Mouse anti-5mC (1:150, abcam, ab10805), Rat anti-BLIMP1 (1:100, eBioscience, 14-5963), Rabbit anti-DNMT1 (1:200, Genetex, GTX116011), Rabbit anti-DNMT3A (1:100, Santa Cruz, sc-20703), Rabbit anti-DNMT3B (1:100, Santa Cruz, sc-20704), Rabbit anti-H3K9me2 (1:500, Millipore, 07-441), Rabbit anti-H3K9me3 (1:500, abcam, ab8898), Rabbit anti-H3K27me3 (1:500, Millipore, 07-449), Mouse anti-HP1α (1:200, Active Motif, 39977), Rabbit anti-Ki67 (1:100, abcam, ab16667), Rabbit anti-KLF4 (1:400, Santa Cruz, sc-20691), Rabbit anti-macroH2A2 (1:500, Active Motif, 39873), Mouse anti-OCT4 (1:500, BD Biosciences, 611203), Goat anti-OCT4 (1:500, Santa Cruz, sc-8629), Goat anti-SOX17 (1:500, R&D Systems, AF1924), Mouse anti-TEAD4 (1:100, abcam, ab58310), Mouse anti-TET1 (1:250, Genetex, GT1462), Rabbit anti-TET2 (1:200,

Genetex, GTX124205), Rabbit anti-TFAP2C (1:200, Santa Cruz, sc-8977), Goat anti-TFCP2L1 (1:500, R&D Systems, AF5726), Mouse anti-UHRF1 (1:200, Active Motif, 61342), Goat anti-VASA (1:500, Active Motif, 61342).

Image Analysis

Quantification of fluorescence signals in confocal images (Figure 3C and 3F) was performed with Volocity 3D Image Analysis Software (PerkinElmer) by a custom workflow. Briefly, each individual nucleus was selected based on DAPI signal. Nuclei which overlaped with OCT4/TFAP2C or surrounded by DDX4 signals were defined as hPGCs, while the rest were defined as neighbouring somatic cells. The fluorescence intensities for the epigenetic modifications or modifiers of interest were then measured in the two populations of nuclei. The distribution of relative intensity was plotted as boxplots. For UHRF1, only nuclei which were positive for KI-67 were included for quantification. Quantification was based on at least 3 confocal images of each stage.

RNA-Seq Library Preparation

Cells were sorted directly into extraction buffer of PicoPure RNA Isolation Kit (Applied Biosystems) and RNA was extracted according to manufacturer's protocol. Total RNA (0.5 to 2 ng) was then reverse transcribed and amplified into cDNA using Ovation RNA-Seq System V2 (Nugen). Amplified cDNA was sonicated into 250 bp by Covaris S220 Focused-ultrasonicators. Subsequently, RNASeq library was generated with 500 ng of fragmented cDNA using Ovation Rapid DR Multiplex System (Nugen). Library was quantified by qPCR using KAPA Library Quantification Kit (Kapa Biosystems) using QuantStudio 6 Flex Real-Time PCR System

(Applied Biosystems). Libraries were subjected to single-end 50 bp sequencing on HiSeq 2000/2500 sequencing system (Illumina). Every 4 indexed libraries were multiplexed to one lane of a flowcell, resulting in >40 millions single end reads per sample.

PBAT Library Preparation

PBAT libraries were constructed as described by Kobayashi et al. (2013) with some modifications. Briefly, hPGCs, gonadal somatic cells or SSEA4-positive conventional H9 ESCs (142 to 5000 cells) (Figure S1F) were incubated with lysis buffer (0.1% SDS, 50 ng/ml carrier RNA (QIAGEN) and 1 mg/ml proteinase K (Zymo Research) in DNase-free water for 60 min at 37°C. Unmethylated lambda phage DNA (0.02-0.1 ng) (Promega) was spiked into the sample before bisulfite treatment with the Methylcode Bisulfite Conversion Kit (Invitrogen) according to the manufacturer's instructions, except that the bisulfite conversion step was increased from 2.5 hours to 3.5 hours. Bisulfite-treated DNA was re-annealed to double-stranded DNA using Klenow fragments (3'–5' exo-) (New England Biolabs) with a 5' biotin tagged primer consisted of an Illumina adaptor followed by 4 random nucleotides at the 3' end (BioPEA2N4: 5'-biotin-ACACTCTTTCCCTACACGACGCTCTTCCGATCTNNNN-3'). The biotinylated first strand molecules were captured using Dynabeads M280 Streptavidin (Invitrogen) and then re-annealed to double-stranded DNA again using Klenow fragments (3'–5' exo-) with random primers containing Illumina adaptors (PE-reverse-N4 for SR sequencing: 5'-CAAGCAGAAGACGGCATACGAGATNNNN-3' and Primer4-N15 for PE sequencing: 5'-CAAGCAGAAGACGGCATACGAGATCGGTCTCGGCATTCCTGCTGAACCGCTCTTCCGATC TNNNNNNNNNNNNNNN-3'). Template DNA strands were then synthesized as cDNA with a second strand (where unmethylated C's were converted to T's) using Phusion Hot Start II High-Fidelity DNA Polymerase (Thermo Scientific) with the Illumina primer PE 1.0 (5'-

AATGATACGGCGACCACCGAGATCTACACTCTTTCCCTACACGACGCTCTTCCGATCT-3’). Depending on the input cell number, 9-12 cycles of amplification was performed using KAPA Library Amplfication Kits with KAPA HiFi DNA Polymerase (Kapa Biosystems). Concentrations of PBAT libraries were determined by qPCR using KAPA Library Quantification Kit (Kapa Biosystems). Libraries were subjected to single-end 100 bp sequencing (except for one ESCs library which was sequenced with paired-end 100bp) on HiSeq 2500 sequencing system (Illumina). Coverage information was summarized in Table S2.

RNA-Seq Analysis

Adapter-and quality-trimmed RNA-seq reads were mapped to the human and mouse reference genomes (UCSC GRCh37/hg19 and GRCm38/mm10) using *TopHat2* (http://ccb.jhu.edu/software/tophat, version: 2.0.13) guided by ENSEMBL 74 gene models. Raw counts per transcripts were obtained using *featureCounts*. Replicates were evaluated, counts were normalized and differential expression of transcripts was evaluated by the *R Bioconductor DESeq* package (www.bioconductor.org). Expression-normalized transcript counts were further normalized by transcript length (per kB). Transcript annotations in all bioinformatics analyses were based on Ensembl (Release 74) considering protein coding, long-noncoding RNA and processed transcripts. Human and mouse orthologs were obtained from Ensembl BioMart (http://www.ensembl.org/info/data/biomart.html). Hierarchical clustering was performed with the R *hclust* functions using the Ward’s method. Principal components were computed by singular value decomposition with the R *princomp* functioned on scaled DESeq-normalized expression levels. Only the 80% most highly expressed transcripts were used for clustering and principal component analysis. Enrichment of Gene Ontology (GO) terms and SMART protein domains in differentially expressed genes was evaluated with the DAVID tool (http://david.abcc.ncifcrf.gov).

For co-expression network analysis, transcriptional regulators with functions in ‘embryonic development’, ‘germ cell development’ and ‘stem cell maintenance’ were manually selected based on their Gene Ontology annotation. A co-expression network of the selected regulators was generated based on all pairwise Pearson correlation coefficients of their gene expression values. Coefficients (> 0.5 and Pvalue < 0.05) were visualized as edges between regulators as nodes using the Cytoscape platform (http://www.cytoscape.org). By selecting the most equivalent time points in human and mouse PGC development, key regulators that were significantly differentially expressed (log2FC > 2 or < -2 and p value < 0.05) between human PGCs (Wk 7) and mouse PGCs (E11.5 to E12.5) or between hPGCLCs and mouse E6.5 to E7.5 PGCs, and that were expressed in one organism and lowly expressed in the other (log2(normalized counts) < 3) at those equivalent developmental time points were compiled and highlighted in the co-expression network.

DNA Methylation Analysis

PBAT-reads were quality-trimmed with *Trim Galore* (http://www.bioinformatics.babraham.ac.uk/projects/trim_galore), and 4 nt random primer sequences at the 5’ end (or 15 nt of read 2 of paired end sequences) and 1 nt at the 3’ end of all reads were removed. PBAT-reads were then mapped to the computationally bisulfite-converted human reference genome (GRCh37/hg19) by using *Bismark* (version: 0.7.12; parameter settings: ‘*-n 2 -l 40*’) tolerating two non-cytosine mismatches in 40 nt regions. Potential PCR duplicates were removed using *samtools rmdup*. Published methylome datasets of Sperm (Molaro et al., 2011), ICM and MII oocyte (Guo et al. 2014) were obtained and mapped in a similar manner as above.

Methylation levels for cytosines (CpGs and non-CpGs) were called with *MethPipe methcounts* (http://smithlabresearch.org/software/methpipe) (Song et al., 2013). Average CpG methylation levels of annotated genomic regions, i.e. of promoters, exons, repeats, imprint control regions, CGIs, enhancers and 1kB tiles, were calculated with the *MethPipe roimethstats* program considering only information from CpGs with >=5X coverage. Only high-confidence genomic regions with at least 5 CpGs covered by >=5X and >20% of their total CpGs covered were used in further analyses. Metagene profiles for CpG methylation levels were generated for using 150 nt-sized windows overlapping all annotated gene regions by using the MethPipe *roimethstats* program and custom PERL and R scripts.

1kB tiles were calculated for all chromosomes with a 500 bp offset. CGI annotations in the human genome were obtained from the UCSC Table Browser. CpG densities were calculated and promoter were classified according to CpG density as described (Weber et al., 2007). Imprint control regions annotation were obtained from Fang et al (2012).

To annotate enhancers, ChIP-seq peak files and uniformly processed and subsampled alignment files for DNaseI hypersensitivity (DHS) and H3K4me1, H3K4me3 and H3K27ac for ESCs derivatives and all *in vivo* cell types were acquired from the Roadmap Epigenomics Project (http://www.broadinstitute.org/~anshul/projects/roadmap) (Kundaje et al., 2015). To identify active enhancer regions, DHS peak regions from all cell types were merged, regions that intersected with H3K4me3 regions marking promoters were discarded, and the remaining merged DHS peaks were intersected with H3K4me1 and H3K27ac peaks to define active enhancers for each cell type.

For comparison of human and mouse PGC methylation dynamics, PBAT reads were downloaded from DDBJ Sequence Read Archive (DRA000607) (Kobayashi et al., 2013) and mapped to the mouse reference genome (GRCm38/mm10) using Bismark. Methylation levels of

cytosines in mouse were compiled using *MethPipe methcounts* program, and methylation levels of all CpGs (>= 5X coverage) were mapped to the human reference genome using the UCSC *liftover* tool. The average methylation levels of 1 kB tiles in the human genome were then calculated using all cytosines regardless of their context (CpG or non-CpG) in the human genome. Only 1kB tiles with >=5X coverage and at least 20% of total human CpGs covered were used for the comparison of human and mouse methylation levels. 1kB tiles were not allowed to intersect with any annotated repeat.

Repeat Expression Analysis

RepeatMasker annotations for the human reference genome were obtained from the UCSC Table Browser. To calculate repeat expression, adapter-trimmed RNA-seq reads were mapped to the human reference genome (GRCh37/hg19) by using *bowtie* (http://bowtie-bio.sourceforge.net; version: 1.1.0) with parameters *'-m1 –v2 –best –strata'* selecting reads that uniquely align to single genomic repeat copies by allowing two mismatches. Read counts for repeat regions and ENSEMBL transcripts were calculated by *featureCounts*, normalized by the total number of RNA-seq reads that mapped to protein-coding gene regions, and subsequently normalized by repeat length. Differential expression of repeat copies across samples was evaluated by the *R Bioconductor DESeq* package. Since total repeat expression was underestimated by rejecting multiply mapping reads, RNA-seq reads were further mapped with *bowtie2* using default parameters to evaluate the average expression of repeat families. In addition, repeats that intersected with exons of annotated long non-coding RNAs (lncRNAs) were evaluated independently by using *TopHat2* and considering the total read counts for the annotated lncRNA transcripts.

Escapee Analysis

To extract regions that are resistant to DNA demethylation, significantly hypermethylated regions (HyperMR) were called in all human Wk7 to Wk9 samples from individual embryos and from pooled samples with *MethPipe* by inverting DNA methylation levels (1-DNA methylation level) at each cytosine. Overlapping HyperMRs were merged using *bedops* tools, and high-confidence regions with at least 20% of their CpGs covered by >= 5X and at least 30% methylation in any of the individual or pooled samples were selected for further analysis. For comparison with mouse, an identical HyperMR analysis was performed for the most de-methylated time points in mouse PGC development E13.5_M and E13.5_F. Overlapping mouse HyperMRs were merged and hyper-methylated regions with at least 30% methylation level were selected. For additional validation, methylation levels of individual CpGs in mouse were mapped to the human reference genome using the UCSC *liftover* tool, and average methylation levels of the HyperMRs identified in human were compiled.

ChIP-seq peak files for H3K9me3, H3K36me3 and H3K79me2 were downloaded for selected cell types from NIH Roadmap Epigenomics Consortium (http://www.broadinstitute.org/~anshul/projects/roadmap) (Kundaje et al., 2015). To calculate the enrichment of epigenetic modifications in escapees, we performed a genome-scale randomization test by generating 1,000 sets of *N* genomic regions with a comparable size distribution and a similar bias towards annotated gene regions as the *N* resistant regions using custom Perl scripts. A one-sided Wilcoxon test was then used to compare the observed value in escapees versus the distribution of values obtained from 1,000 randomly generated sets of genomic regions. DNA binding specificities for KRAB zinc finger proteins were predicted *de novo* based on randomized C2H2-ZF library screens (Persikov et al., 2015), escapees were screened for the motif using *FIMO*, and the enrichment of KRAB zinc finger binding sites was evaluated against 1,000 random genomic regions. Tissue-specific expression according to the

manually curated UniProt annotation (UP_TISSUE) and enrichment of Gene Ontology (GO) terms, KEGG pathways and SMART protein domains was evaluated with the DAVID tool. To trace the fate of escapees, k-means clustering was performed on common repeat-poor escapees of Wk7-Wk9 hPGC, with recently published high coverage PBAT dataset of human oocytes, sperm and blastocyst (Okae et al. 2014).

SUPPLEMENTAL REFERENCES

Fang, F., Hodges, E., Molaro, A., Dean, M., Hannon, G.J., and Smith, A.D. (2012). Genomic landscape of human allele-specific DNA methylation. Proceedings of the National Academy of Sciences of the United States of America *109*, 7332-7337.

Kundaje, A., Meuleman, W., Ernst, J., Bilenky, M., Yen, A., Heravi-Moussavi, A., Kheradpour, P., Zhang, Z., Wang, J., Ziller, M.J., *et al.* (2015). Integrative analysis of 111 reference human epigenomes. Nature *518*, 317-330.

Persikov, A.V., Wetzel, J.L., Rowland, E.F., Oakes, B.L., Xu, D.J., Singh, M., and Noyes, M.B. (2015). A systematic survey of the Cys2His2 zinc finger DNA-binding landscape. Nucleic acids research *43*, 1965-1984.

Song, Q., Decato, B., Hong, E.E., Zhou, M., Fang, F., Qu, J., Garvin, T., Kessler, M., Zhou, J., and Smith, A.D. (2013). A reference methylome database and analysis pipeline to facilitate integrative and comparative epigenomics. PloS one *8*, e81148.

Weber, M., Hellmann, I., Stadler, M.B., Ramos, L., Paabo, S., Rebhan, M., and Schubeler, D. (2007). Distribution, silencing potential and evolutionary impact of promoter DNA methylation in the human genome. Nature genetics *39*, 457-466.

Table S4. Glu-qPCR Primer Sequences, Related to Figure 3 and Extended Experimental Procedures

**Loci**		**Primer Sequence 5'-3'**
GNAS ICR	F	AGACCGAGCCTGAAGACGAT
	R	CAACTTGAGAGCGTGCAGAC
H19 ICR	F	CCTATACCTCACGACCCCTGT
	R	CTCACACATCACAGCCTGAGC
DAZL Promoter	F	CTCTCCCTCAACTCACCATGA
	R	CACAGCAGCCCCAGAAGT
DDX4 Promoter	F	ATGAGCCTCAGCTGCACTTT
	R	CTCTCCCCTTTTACCCATCAC
Chr12 Control	F	GGTCATGAATGCTTCTGAGGA
	R	GGCTGTGCTGACTTGAGAACT
