# Supplementary material for: A Unique Gene Regulatory Network Resets the Human Germline Epigenome for Development
Source: Cell. 2015 Jun 4;161(6):1453–67. doi: 10.1016/j.cell.2015.04.053 (PMC4459712; doi:10.1016/j.cell.2015.04.053)
Supplement: Document S2. Article plus Supplemental Information [file mmc5.pdf]

Resource

Cell

A Unique Gene Regulatory Network Resets the Human Germline Epigenome for Development

Graphical Abstract

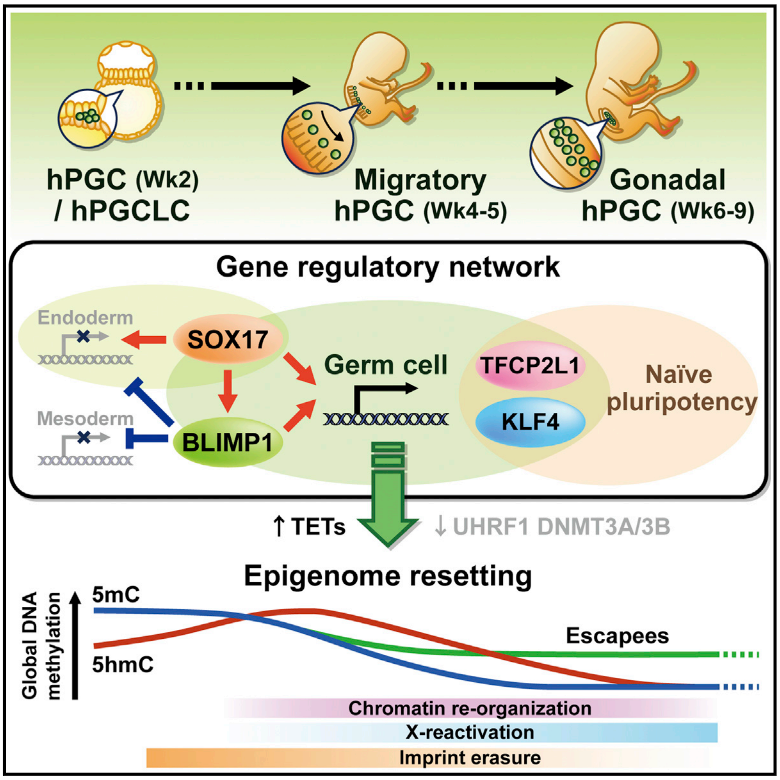

Highlights

- SOX17-BLIMP1 with TFCP2L1 and KLF4 constitute a unique hPGC transcriptome
- hPGC transcriptome drives extensive DNA demethylation and chromatin reorganization
- Evolutionarily young and hazardous retrotransposons remain partially methylated
- Some demethylation resistant loci are candidates for epigenetic inheritance

Authors

Walfred W.C. Tang, Sabine Dietmann, ..., Patrick F. Chinnery, M. Azim Surani

Correspondence

a.surani@gurdon.cam.ac.uk

In Brief

A unique transcriptome drives extensive epigenome resetting in human primordial germ cells for establishment of totipotency. Some loci associated with metabolic and neurological disorders exhibit resistance to reprogramming and are candidates for transgenerational epigenetic inheritance.

Tang et al., 2015, Cell *161*, 1453–1467
June 4, 2015 ©2015 The Authors
http://dx.doi.org/10.1016/j.cell.2015.04.053

Cell

A Unique Gene Regulatory Network Resets the Human Germline Epigenome for Development

Walfred W.C. Tang,[1,2,3,5] Sabine Dietmann,[3,5] Naoko Irie,[1,2,3] Harry G. Leitch,[3] Vasileios I. Floros,[4] Charles R. Bradshaw,[1] Jamie A. Hackett,[1,2,3] Patrick F. Chinnery,[4] and M. Azim Surani[1,2,3,*]
[1]Wellcome Trust Cancer Research UK Gurdon Institute, Tennis Court Road, University of Cambridge, Cambridge CB2 1QN, UK
[2]Department of Physiology, Development and Neuroscience, Downing Street, University of Cambridge, Cambridge CB2 3EG, UK
[3]Wellcome Trust-Medical Research Council Stem Cell Institute, Tennis Court Road, University of Cambridge, Cambridge CB2 3EG, UK
[4]Wellcome Centre for Mitochondrial Research, Institute of Genetic Medicine, Newcastle University, Newcastle upon Tyne NE1 3BZ, UK
[5]Co-first author
*Correspondence: a.surani@gurdon.cam.ac.uk
http://dx.doi.org/10.1016/j.cell.2015.04.053
This is an open access article under the CC BY license (http://creativecommons.org/licenses/by/4.0/).

SUMMARY

Resetting of the epigenome in human primordial germ cells (hPGCs) is critical for development. We show that the transcriptional program of hPGCs is distinct from that in mice, with co-expression of somatic specifiers and naive pluripotency genes TFCP2L1 and KLF4. This unique gene regulatory network, established by SOX17 and BLIMP1, drives comprehensive germline DNA demethylation by repressing DNA methylation pathways and activating TET-mediated hydroxymethylation. Base-resolution methylome analysis reveals progressive DNA demethylation to basal levels in week 5–7 in vivo hPGCs. Concurrently, hPGCs undergo chromatin reorganization, X reactivation, and imprint erasure. Despite global hypomethylation, evolutionarily young and potentially hazardous retroelements, like SVA, remain methylated. Remarkably, some loci associated with metabolic and neurological disorders are also resistant to DNA demethylation, revealing potential for transgenerational epigenetic inheritance that may have phenotypic consequences. We provide comprehensive insight on early human germline transcriptional network and epigenetic reprogramming that subsequently impacts human development and disease.

INTRODUCTION

The epigenome is extensively reprogrammed in the mammalian germline and in preimplantation embryos. Epigenetic reprogramming during preimplantation development resets the gametic epigenome for naive pluripotency (Guo et al., 2014; Smith et al., 2014), whereas reprogramming in primordial germ cells (PGCs), which includes erasure of genomic imprints and potentially epimutations, restores full germline potency for the transmission of genetic and epigenetic information (Hajkova et al., 2002). Recent studies on preimplantation embryos have provided some insights on this process in humans, but our knowledge of the human germline remains imprecise.

Mouse is the key mammalian model for germline studies. Aligning early embryological events between mice and humans is informative for human germline biology (Figure 1A) (Leitch et al., 2013). Human PGCs (hPGCs) are specified at approximately embryonic day (E) 12–16 (developmental week [Wk] 2) in the posterior epiblast of early postimplantation embryos, compared to E6.25 in mice. At Wk3–Wk5 (analogous to E8–E10.5 in mice), hPGCs migrate from the yolk sac wall through the hindgut and colonize the developing genital ridge. Following extensive proliferation, female hPGCs enter meiosis asynchronously after Wk9, whereas mPGCs do so synchronously at E13.5. However, male germ cells of both species enter mitotic quiescence and undergo meiosis after puberty. Thus, Wk2–Wk9 hPGCs can be aligned with E6.25–E13.5 mPGCs (Figure 1A).

Using our newly developed in vitro model for hPGC-like cell (hPGCLC) specification, we discovered that SOX17 is the key specifier of human germ cell fate, whereas BLIMP1 acts in tandem to repress mesendoderm differentiation (Irie et al., 2015). In contrast, SOX17 is dispensable in mPGCs, where BLIMP1, PRDM14, and TFAP2C are critical regulators (Magnúsdóttir et al., 2013; Nakaki et al., 2013). This fundamental mechanistic difference for PGC specification has implications for the launch of epigenetic reprogramming, as the transcriptional and epigenetic programs are intimately linked.

In mice, global epigenome resetting occurs as mPGCs migrate and colonize the genital ridge (E8–E13.5) (Figure 1A). Following repression of DNA methylation pathways, genome-wide loss of 5-methylcytosine (5mC) occurs through replication-coupled dilution (Guibert et al., 2012; Kagiwada et al., 2013; Seisenberger et al., 2012) and by conversion of 5mC to 5-hydroxymethylcytosine (5hmC) by TET enzymes (Dawlaty et al., 2013; Hackett et al., 2013; Yamaguchi et al., 2013). Concomitantly, X reactivation and chromatin reorganization, including depletion of H3K9me2 and enrichment of H3K27me3, also occur in mPGCs (Chuva de Sousa Lopes et al., 2008; Seki et al., 2005), leading to a basal epigenetic state at ~E13.5. Nonetheless, DNA methylation persists at specific loci in mPGCs, with a potential for epigenetic

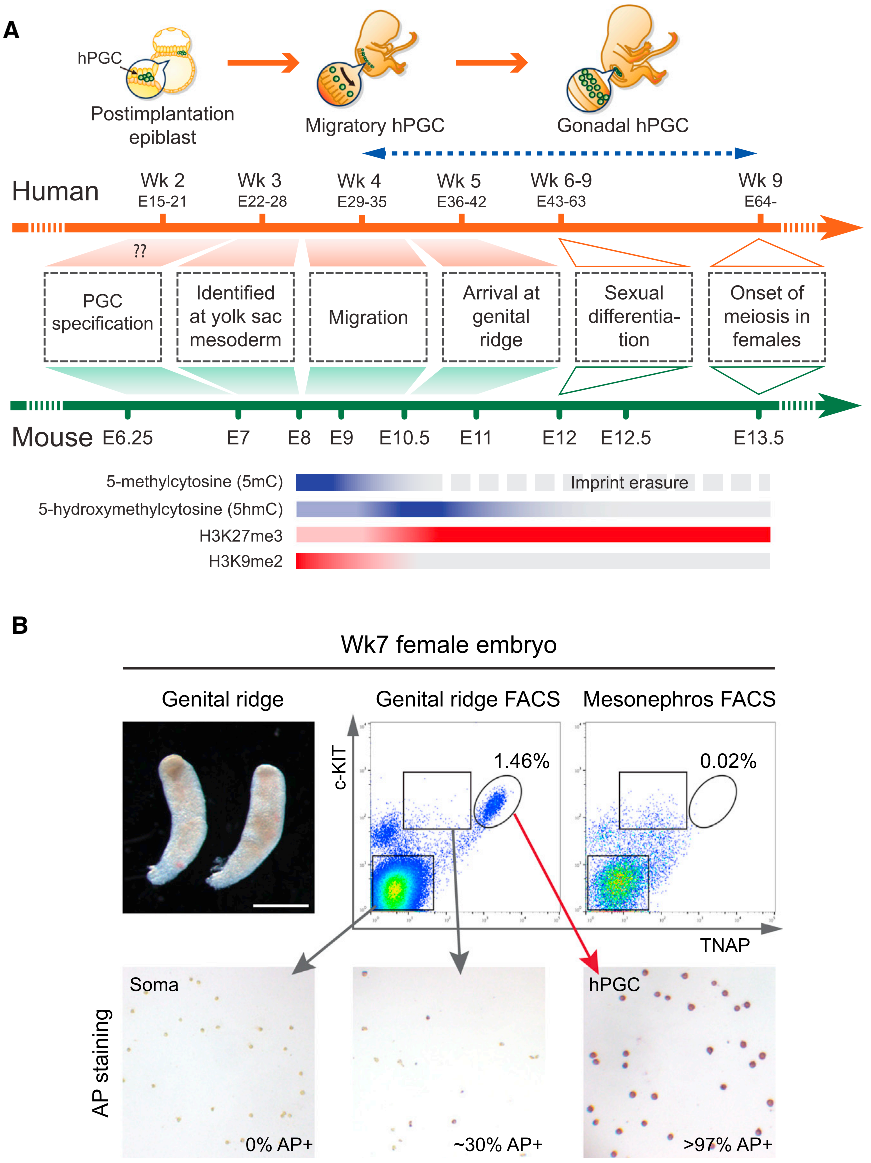

Figure 1. Developmental Timeline and Isolation of a Pure Population of hPGCs

(A) Developmental timelines of human and mouse PGCs based on embryological landmarks of germ cell development. Notable epigenetic changes in mPGCs are depicted as colored bars. Blue arrow line indicates developmental ages of human embryos covered in the current study.

(B) Isolation of hPGCs from Wk7 female embryonic gonads by FACS with cell-surface markers TNAP and c-KIT. Mesonephros is used as a negative control. The purity of hPGCs was tested by alkaline phosphatase staining (bottom).

See also Figure S1.

inheritance (Hackett et al., 2013; Seisenberger et al., 2012). Global depletion of DNA methylation in hPGCs apparently occurs by Wk10 (Gkountela et al., 2013), but the precise demethylation dynamics at the earlier critical stages are largely unknown.

Here, we studied transcriptome transitions and epigenetic reprogramming in Wk4–Wk9 in vivo hPGCs and nascent hPGCLCs by RNA-sequencing (RNA-seq) and whole-genome bisulfite sequencing (BS-seq). We found that hPGCs acquire a transcriptional program that is distinct from the mouse germline. Under this unique gene regulatory network, DNA methylation pathways are suppressed while TET-mediated hydroxymethylation is activated. This leads to comprehensive DNA demethylation and chromatin reorganization in Wk4–Wk9 hPGCs. Despite global hypomethylation, resistance to DNA demethylation was observed in some retrotransposon-associated and single copy regions, which are potential mediators of epigenetic memory and transgenerational inheritance in humans. Our study presents an important advance on the epigenetic and transcriptional programs of the human germline.

RESULTS

Isolation of a Pure Population of hPGCs

With ethical approval, we obtained Wk4–Wk9 human embryos to investigate hPGC development (Figure S1A). First, we established a fluorescence-activated cell sorting (FACS) protocol to isolate hPGCs from genital ridges. Using cell-surface markers TNAP (tissue non-specific alkaline phosphatase) and c-KIT, we consistently obtained hPGCs of high purity, with >97% of cells from the unique TNAP-high and c-KIT-high population positive for alkaline phosphatase (AP) staining (Figures 1B and S1B, see also transcription profile in Figure 2C). In contrast, only ~30% of the TNAP-medium and c-KIT-high cells were AP positive, and such a population was also found in mesonephros, which is devoid of hPGCs (Figure 1B). This suggests that isolation of hPGCs by c-KIT alone as previously reported (Gkountela et al., 2013) might not yield a pure hPGC population.

We performed RNA-seq and BS-seq on purified hPGCs (TNAP-high and c-KIT-high) and gonadal somatic cells (TNAP and c-KIT double negative) from 14 individual human embryos of Wk5.5, Wk7, and Wk9 (Figure S1C). These stages cover mitotic hPGCs immediately after colonization of the genital ridges, before meiotic entry in oogonia and mitotic quiescence in prospermatogonia (Figure 1A).

RNA-Seq Reveals Unique Transcriptional States of hPGCs

Unsupervised hierarchical clustering of RNA-seq transcription profiles showed that Wk5.5–Wk9 hPGCs samples formed one distinct branch, away from conventional primed H9 ESCs and

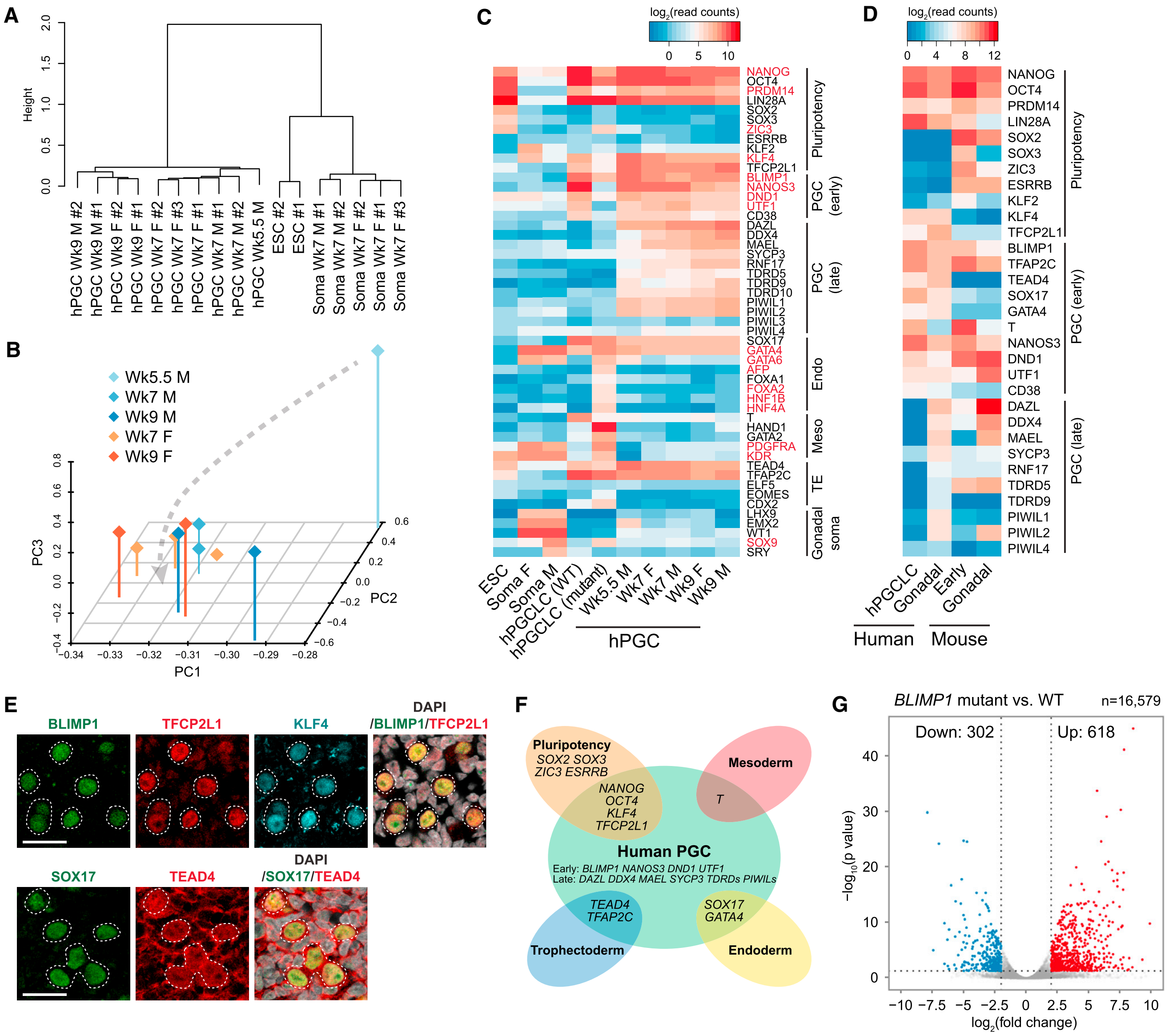

Figure 2. RNA-Seq Reveals Unique Transcriptional States of hPGCs

(A) Hierarchical clustering of gene expression profiles. Biological replicates of Wk5.5–Wk9 male (M) and female (F) hPGCs, gonadal somatic cells (soma), and conventional H9 ESCs are shown. Note that only one Wk5.5 hPGC sample was available for RNA-seq.

(B) Principal component analysis (PCA) of gene expression in hPGC samples. Arrow line indicates developmental progression along PC2 and PC1.

(C) Heatmap showing mean expression of representative genes in human samples. Differentially expressed genes between day 4 wild-type (WT) and *BLIMP1* mutant hPGCLCs [$\log_2$(fold change)>2, $p < 0.05$] are highlighted. Note that mutant cells lack BLIMP1 protein as determined by immunofluorescence, but frame-shifted mutant transcripts are detected. "Endo," endoderm; "Meso," mesoderm; "TE," trophectoderm.

(D) Expression of key genes in human and mouse PGCs. Mean expression in biological replicates of hPGCLCs, Wk7–Wk9 hPGCs (gonadal), E7.5 (early), and E11.5–12.5 (gonadal) mPGCs are shown.

(E) Immunofluorescence of TFCP2L1, KLF4, and TEAD4 on Wk7 female genital ridge cryosections. hPGCs are counterstained by BLIMP1 or SOX17. Scale bars, 20 μm.

(F) Schematic illustrating the unique transcriptome of hPGCs.

(G) Volcano plot showing differentially expressed genes between day 4 *BLIMP1* mutant and wild-type (WT) hPGCLCs [$\log_2$(fold change)>2, $p < 0.05$].

See also Figure S2 and Table S1.

gonadal somatic cells (referred to as "soma") (Figure 2A). Interestingly, Wk5–Wk9 hPGCs clustered according to their stage of development, without prominent difference between male and female germ cells. Principal component analysis (PCA) showed developmental progression along PC2 (Figure 2B), with meiotic markers, such as *DDX4*, *SYCP2*, and *TEX14*, loaded more heavily to the lower end of PC2 (Figure S2A). Gene ontology (GO) analysis of upregulated genes in Wk9 over Wk5.5 hPGCs

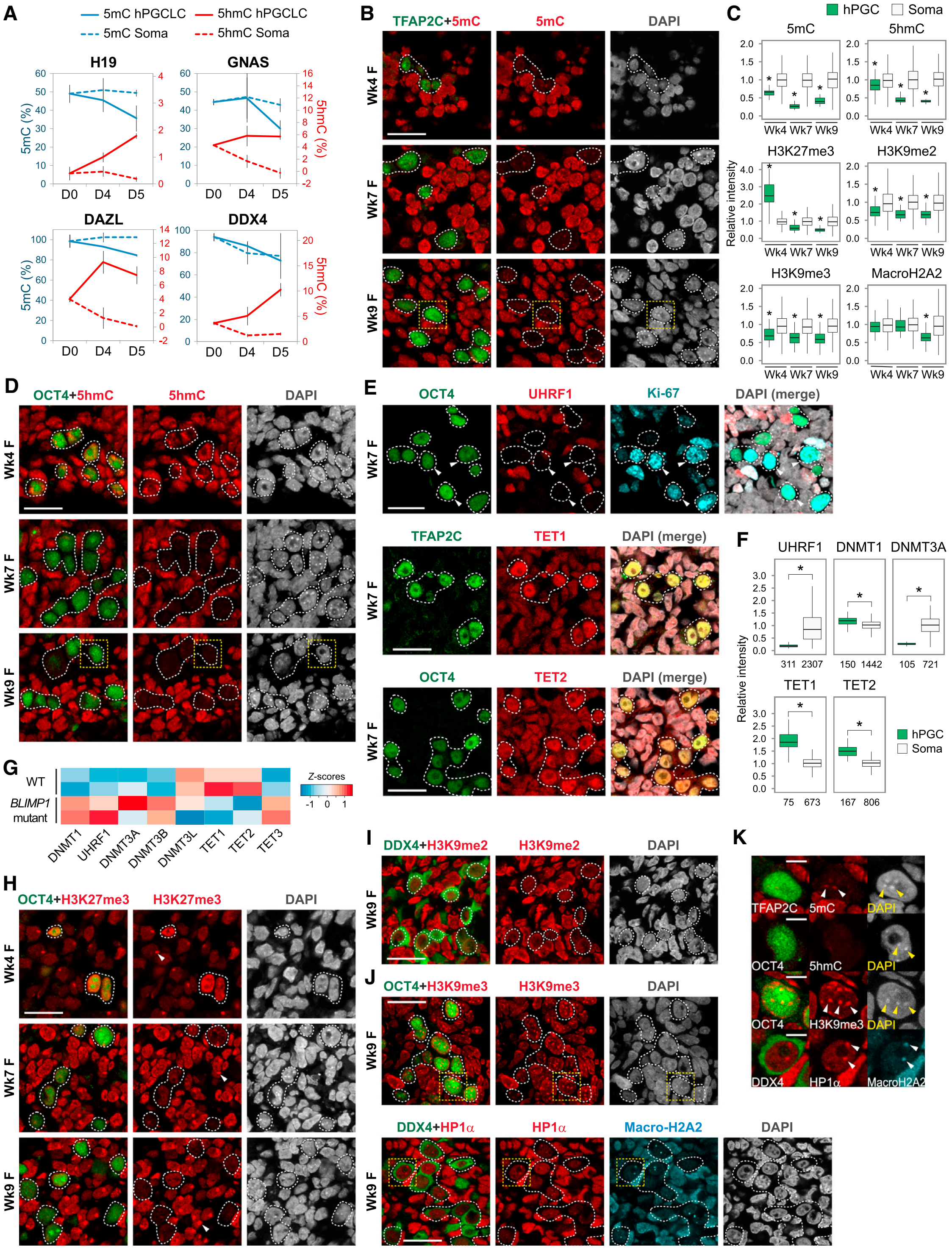

Figure 3. DNA Demethylation and Chromatin Reorganization in the Human Germline

(A) 5mC and 5hmC levels at ICRs of *H19* and *GNAS* ICRs and promoters of *DAZL* and *DDX4* in hPGCLCs and surrounding soma determined by Glu-qPCR. Day (D) 0 represents 4i ESCs. Data are represented as mean ± SEM of two biological replicates.

(B and D) Immunofluorescence analysis for (B) 5-methylcytosine (5mC) and (D) 5-hydroxymethylcytosine (5hmC) on human embryo cryosections. hPGCs are counterstained by TFAP2C or OCT4. Scale bars, 20 μm.

(C) Fluorescence intensity of indicated epigenetic modifications in hPGCs and surrounding soma (corresponds to images in Figures 3B, 3D, 3H–3J, S3E, and S3F). Around 20–800 hPGCs and >200 soma are used for quantification at each time point. (*$p < 0.01$ between hPGC and soma; Wilcoxon signed-rank test.)

(legend continued on next page)

also showed enrichment of biological process terms, such as “male meiosis” and “piRNA metabolic process” (Figure S2B). Indeed, late PGC genes were increasingly expressed from Wk5.5 to Wk9 (Figure 2C and Table S1), suggesting progressive germ cell differentiation after colonization of the genital ridges. Expression of sexual differentiation genes *LHX9*, *EMX2*, *WT1*, and *GATA6* was already evident in Wk7 soma, with *SOX9* and *SRY* specifically in males (Figure 2C). Importantly, the absence of these somatic genes in hPGCs confirmed their high purity.

Next, we compared human and mouse PGCs transcriptome (Magnúsdóttir et al., 2013), including day 4 hPGCLCs to represent nascent human germ cells (Irie et al., 2015). Correlation of global gene expression suggested that Wk7–Wk9 hPGCs were most similar to gonadal mPGCs at E11.5 to E12.5, whereas hPGCLCs correlated to pre-migratory mPGCs at E6.5–E7.5 (Figure S2C). As revealed by gene co-expression network analysis, human and mouse PGCs shared a core transcriptome consisting of key germ cell genes (e.g., *BLIMP1*, *TFAP2C*, *UTF1*, *DAZL*, and *DDX4*), as well as pluripotency genes (e.g., *OCT4*, *NANOG*, *PRDM14*, and *LIN28A*) (Figures 2D and S2D). However, there were some significant differences between the two species. For example, key pluripotency factors *ESRRB*, *SOX2*, *SOX3*, and *ZIC3*, which are strongly expressed in mPGCs, were absent in hPGCs and hPGCLCs (Figure 2D). In contrast, hPGCs highly expressed *KLF4* and *TFCP2L1* (Figures 2D and 2E), which are detected in inner cell mass (ICM) and ground state human ESCs, but not in primed ESCs (Takashima et al., 2014). Endoderm specifiers *SOX17* and *GATA4* were also detected, with SOX17 being indispensable for hPGCLC specification (Irie et al., 2015). Furthermore, hPGCs and hPGCLCs co-expressed *TEAD4* (Figures 2C–2E), a trophectoderm specifier that is absent in mPGCs (Sasaki, 2010). Thus, the hPGC transcriptome is composed of core germ cell genes with somatic and trophectoderm lineage specifiers and naive pluripotency genes (Figure 2F). This regulatory network is distinct from that of mice and unlike the classic pluripotency circuitry.

In hPGCs, we also detected the expression of *human endogenous retrovirus subfamily H* (*HERVH*) (Figure S2E), an abundantly expressed long non-coding RNA (lncRNA) in ESCs that associates with OCT4 to regulate the human pluripotency network (Lu et al., 2014). However, *HERVH* in hPGCs originated from genomic loci distinct from that of ESCs. The function of these hPGC-specific HERVH elements (cluster 3) remains to be determined, but this result further indicates that the pluripotency network in hPGCs is rewired and is distinct from that in ESCs (Figure 2F).

SOX17 and BLIMP1 are important for hPGCLC specification (Irie et al., 2015). While loss of SOX17 abolished human germline establishment, *BLIMP1* mutant hPGCLCs failed to turn on NANOS3-mCherry reporter and appeared as TNAP-single positive cells at day 4, which disappeared by day 8. To further elucidate the contribution of BLIMP1 to the germline transcriptome, we performed RNA-seq on day 4 mutant hPGCLCs. We found 920 differentially expressed genes between wild-type and mutant cells, with 618 (68%) of them being derepressed (Figure 2G). In particular, mutant hPGCLCs aberrantly expressed endoderm (e.g., *GATA6* and *AFP*), mesoderm (e.g., *GATA2* and *KDR*), and HOX genes, whereas early PGC (e.g., *NANOS3* and *UTF1*) and pluripotency (e.g., TFCP2L1 and *KLF4*) genes were downregulated (Figure 2C and Table S1). However, mutant cells still expressed other notable hPGC genes, including *SOX17*, *TFAP2C*, *TEAD4*, and *OCT4*, albeit at lower levels. Thus, BLIMP1 suppresses mesendoderm genes induced by upstream BMP signaling and SOX17 (Irie et al., 2015) and acts in tandem with other factors to establish the unique human germ cell transcriptional program.

Resetting the Human Germline Methylome

Following upregulation of SOX17 and BLIMP1, maintenance and de novo DNA methylation pathways are repressed in hPGCLCs, whereas 5hmC is globally enriched with upregulation of TET1 (Irie et al., 2015). Because erasure of genomic imprints is a unique hallmark of PGC reprogramming (Hajkova et al., 2002), we examined the methylation status of imprint control regions (ICRs) in day 4 and 5 nascent hPGCLCs. Even at this early germline stage, we observed partial erasure of 5mC coupled with 5hmC enrichment at *H19* and *GNAS* ICRs (Figure 3A). Notably, the promoters of late germ cell genes *DAZL* and *DDX4* were also targeted for hydroxymethylation and exhibited partial 5mC reduction (Figure 3A), although they still retained >80% 5mC and remained repressed (Figure 2C). This indicates the initiation of methylome resetting in nascent hPGCLCs via oxidation of 5mC to 5hmC by TET enzymes.

As hPGCLCs under current conditions do not develop further in vitro, we followed downstream reprogramming events in hPGCs of Wk4–Wk9 human embryos. We found that Wk4 migratory hPGCs at the hindgut already exhibited low global 5mC compared to surrounding soma, and Wk7-Wk9 gonadal hPGCs remained devoid of 5mC (Figures 3B and 3C). In contrast, 5hmC was detectable in hPGC as bright foci at some nuclear regions at Wk4 but became gradually depleted by Wk9 (Figures 3C and 3D). Consistent with global demethylation, UHRF1, which targets DNMT1 to replication foci for maintenance DNA methylation, was not detectable in proliferating hPGCs (Figures 3E, 3F, S3A, and S3D). De novo DNA methyltransferase DNMT3A and DNMT3B were also repressed (Figures 3F and S3B),

(E) Immunofluorescence analysis for UHRF1, TET1, and TET2 on genital ridge. Arrowheads indicate Ki-67-positive (proliferating) hPGCs, which are UHRF1 negative. Scale bars, 20 μm.

(F) Fluorescence intensity of epigenetic modifiers in Wk7 female hPGCs and surrounding soma (corresponds to images in Figures 3E, S3A, and S3B). Sample sizes are indicated. (*$p < 0.0001$; Wilcoxon signed-rank test.)

(G) Expression of epigenetic modifiers in biological replicates of day 4 wild-type (WT) and *BLIMP1* mutant hPGCLCs by RNA-seq.

(H–J) Immunofluorescence analysis for (H) H3K27me3, (I) H3K9me2, (J) H3K9me3, and HP1α/ MacroH2A2. Arrowheads in (H) indicate H3K27me3 foci in somatic cells. Scale bars, 20 μm.

(K) Magnified immunofluorescence images (corresponds to yellow boxes in Figures 3B, 3D, and 3J) showing hPGCs with enrichment of 5mC, H3K9me3, and MacroH2A2 at chromocenters (arrowheads), which is not the case for 5hmC. Scale bars, 5 μm.

See also Figure S3.

whereas TET1 and TET2 were enriched (Figures 3E, 3F, and S3C). As no maintenance mechanism of 5hmC is yet known in mammals, conversion of 5mC to 5hmC likely resulted in passive dilution during replication (Kagiwada et al., 2013). We did not detect 5-formylcytosine (5fC) and 5-carboxylcytosine (5caC), the 5hmC downstream oxidation products (data not shown), or significant levels of thymine DNA glycosylase (*TDG*) (Figure S3D). These hPGC in vivo demethylation dynamics aligns with epigenetic events in E9.5–E13.5 mPGCs (Figure 1A) (Hackett et al., 2013; Seki et al., 2005) and are coherent with the initiation of epigenome resetting in nascent hPGCLCs.

BLIMP1 is a critical contributor to initiation of epigenetic reprogramming in mPGCs by repression of DNA methylation pathways (Magnúsdóttir et al., 2013). We therefore explored its potential role in regulation of the epigenetic program in humans. Compared to wild-type hPGCLCs, loss of *BLIMP1* resulted in higher expression of maintenance and de novo DNA methylation genes, particularly *DNMT3B* (Figure 3G). Furthermore, there was relative reduction of *TET1* and *TET2*, with *TET3* being aberrantly expressed. Loss of BLIMP1 might therefore affect initiation of DNA demethylation. The continual expression of BLIMP1 with upstream regulator SOX17 in Wk4–Wk9 hPGCs likely sustains the epigenetic program toward comprehensive 5mC erasure.

Chromatin Re-organization Occurs in Wk4–Wk9 hPGCs

We next examined the chromatin dynamics in Wk4-9 hPGCs. Post-migratory mPGCs show extensive depletion of H3K9me2 and persistent enrichment of H3K27me3 (Figure 1A) (Seki et al., 2005). In hPGCs, H3K9me2 levels were persistently lower compared to soma, but it remained detectable in the nuclei (Figures 3C, 3I, and S3E). Although Wk4 hPGCs showed 2.5-fold enrichment of H3K27me3, the signal diminished gradually to half of that of soma by Wk9 (Figures 3C and 3H). Thus, human and mouse PGCs demonstrate distinct H3K9me2 and H3K27me3 dynamics. Importantly, H3K27me3 foci at the inactivated X chromosome (Xi) were observed in soma but not in Wk7 female hPGCs (Figure 3H), indicating reactivation of Xi as in mPGCs (Chuva de Sousa Lopes et al., 2008).

Furthermore, we observed global reduction of heterochromatin-associated H3K9me3, heterochromatin protein 1α (HP1α), and macroH2A2 in Wk4-Wk9 hPGCs (Figures 3C, 3J, and S3F). Despite the low global levels, these repressive marks were particularly enriched at DAPI-dense pericentric heterochromatin foci (Figure 3K). Careful examination revealed retention of 5mC but not 5hmC at DAPI-dense chromocenters in Wk9 hPGC (Figure 3K). In contrast, chromocenters are targeted for hydroxymethylation in mPGCs (Hackett et al., 2013; Yamaguchi et al., 2013). This shows that constitutive heterochromatin identity is particularly maintained in hPGCs upon global DNA hypomethylation.

Comprehensive DNA Demethylation in hPGCs Revealed by Base-Resolution BS-Seq

To gain further insights on human germline reprogramming, we studied hPGC methylome at base resolution. We typically obtained ~150 hPGCs per embryo at Wk5.5 and up to 20,000 at Wk7–Wk9 (Figures S1C and S1D). Because reduced representation bisulfite sequencing (RRBS) covers only 5%–10% CpGs with bias toward high-CpG-density regions (Guo et al., 2014; Smith et al., 2014), we adopted the post-bisulfite adaptor tagging (PBAT) method, which allows whole-genome bisulfite sequencing with picogram level of input DNA (Kobayashi et al., 2013; Miura et al., 2012).

We made PBAT libraries with Wk5.5, Wk7, and Wk9 female and male hPGCs, with two biological replicates (two individual embryos) per stage. This yielded BS-seq datasets with up to 82% of total genomic CpG sites covered by at least one read (1×) and 35% by at least 5× per replicate (Table S2). Unmethylated lambda phage DNA spiked into the PBAT libraries revealed bisulfite conversion rates of >99.5%. As biological replicates showed high reproducibility (Figure S4A), we pooled replicates data to further increase coverage up to 56% of CpGs covered by at least 5× per stage (Table S2).

Consistent with the immunofluorescence analysis (Figures 3B and 3D), Wk5.5 female hPGCs were globally hypomethylated with median CpG methylation of 1 kb genomic tiles at 16%, which declined further to a basal level of 4.5% by Wk7 and remained low at Wk9 in both female and male hPGCs (Figure 4A). In contrast, Wk7 female gonadal somatic cells (soma) and conventional H9 ESCs showed >79% DNA methylation. Pair-wise comparisons of changes in methylation suggested largely unidirectional demethylation dynamics between ESCs and Wk5.5 hPGCs, with the vast majority of 1 kb probes losing >60% of methylation (Figure 4C). Most of the partially methylated tiles in Wk5.5 hPGCs also became demethylated by Wk7.

To gain insights across the entire human germline cycle, we included published BS-seq data of human sperm and ICM (Guo et al., 2014; Molaro et al., 2011). As a result of de novo methylation, sperm dramatically gained methylation to 87% from ~4% in Wk9 male hPGCs (Figures 4A and 4C). Despite global DNA demethylation in human pre-implantation embryos (Guo et al., 2014; Smith et al., 2014), ICM retained ~37% methylation (Figure 4A), with the majority of methylated probes in ICM demethylated in hPGCs (Figure 4C). Indeed, hPGCs underwent a more comprehensive wave of epigenome resetting over all genomic features, including CpG islands (CGI), promoters of various CpG densities, exons, introns, intergenic regions, and enhancers (Figures 4B and S4B), regardless of CpG density (Figure S4C).

Nonetheless, a larger fraction (~10%) of repetitive-sequence-containing tiles retained >20% CpG methylation compared to repeat-free tiles (~3%) (Figure S4D), indicating that repetitive elements were more resistant to demethylation (see Figure 6). K-means clustering of repeat-free tiles also revealed a cluster that retained partial methylation at all stages of hPGCs (Figure 4D).

Thus, development of first trimester hPGCs represents the most comprehensive wave of in vivo DNA demethylation known so far in humans. Despite this global epigenetic resetting, a small fraction of unique and repetitive regions escapes global demethylation (see Figures 6 and 7).

Methylation Dynamics of Human versus Mouse PGCs

To compare methylomes of human and mouse PGCs, we lifted over published mPGCs BS-seq data (Kobayashi et al., 2013) to the human genome by sequence conservation and compared

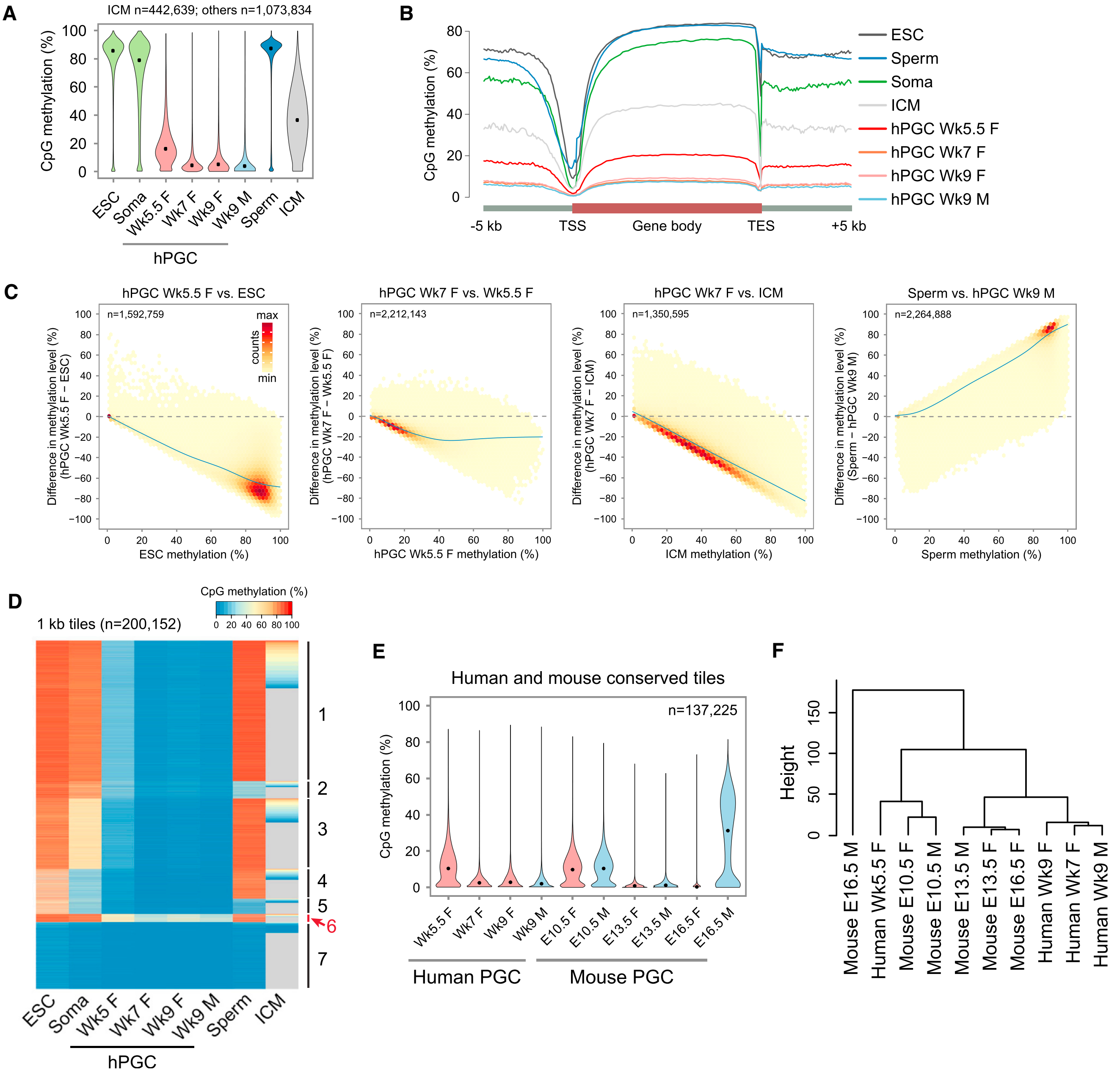

Figure 4. Comprehensive DNA Demethylation in hPGCs Revealed by Base-Resolution BS-Seq

(A) Violin plots showing distribution of CpG methylation levels in overlapped 1 kb genomic tiles of conventional H9 ESCs, Wk7 female gonadal somatic cells (soma), Wk5.5–Wk9 female and male hPGCs, sperm, and ICM. Common tiles with a minimum of 5 CpGs and at least 20% of the total CpGs covered by at least 5× in each sample are considered. These thresholds were applied to all subsequent methylation analyses unless stated otherwise. Due to low coverage, ICM only has ~42% of common tiles fulfilling the above criteria. Black point indicates median.

(B) Averaged CpG methylation level profiles of all genes from 5 kb upstream (−) of transcription start sites (TSSs), through scaled gene bodies to 5 kb downstream (+) of transcription end sites (TES).

(C) Density plots illustrating DNA methylation dynamics of 1 kb tiles between indicated pairs of samples. Color intensity indicates tile counts within each bin, whereas blue regression lines show trends of methylation changes.

(D) K-means clustering of repeat-free tiles into seven dynamic groups. Gray tiles of ICM do not pass coverage thresholds and are therefore not shown. Tiles in cluster 6 retain partial methylation in hPGCs.

(E) Violin plots showing distribution of CpG methylation levels in human and mouse PGCs at conserved repeat-free 1 kb tiles.

(F) Unsupervised hierarchical clustering of methylation levels of conserved repeat-free 1 kb tiles in human and mouse PGCs.

See also Figure S4 and Table S2.

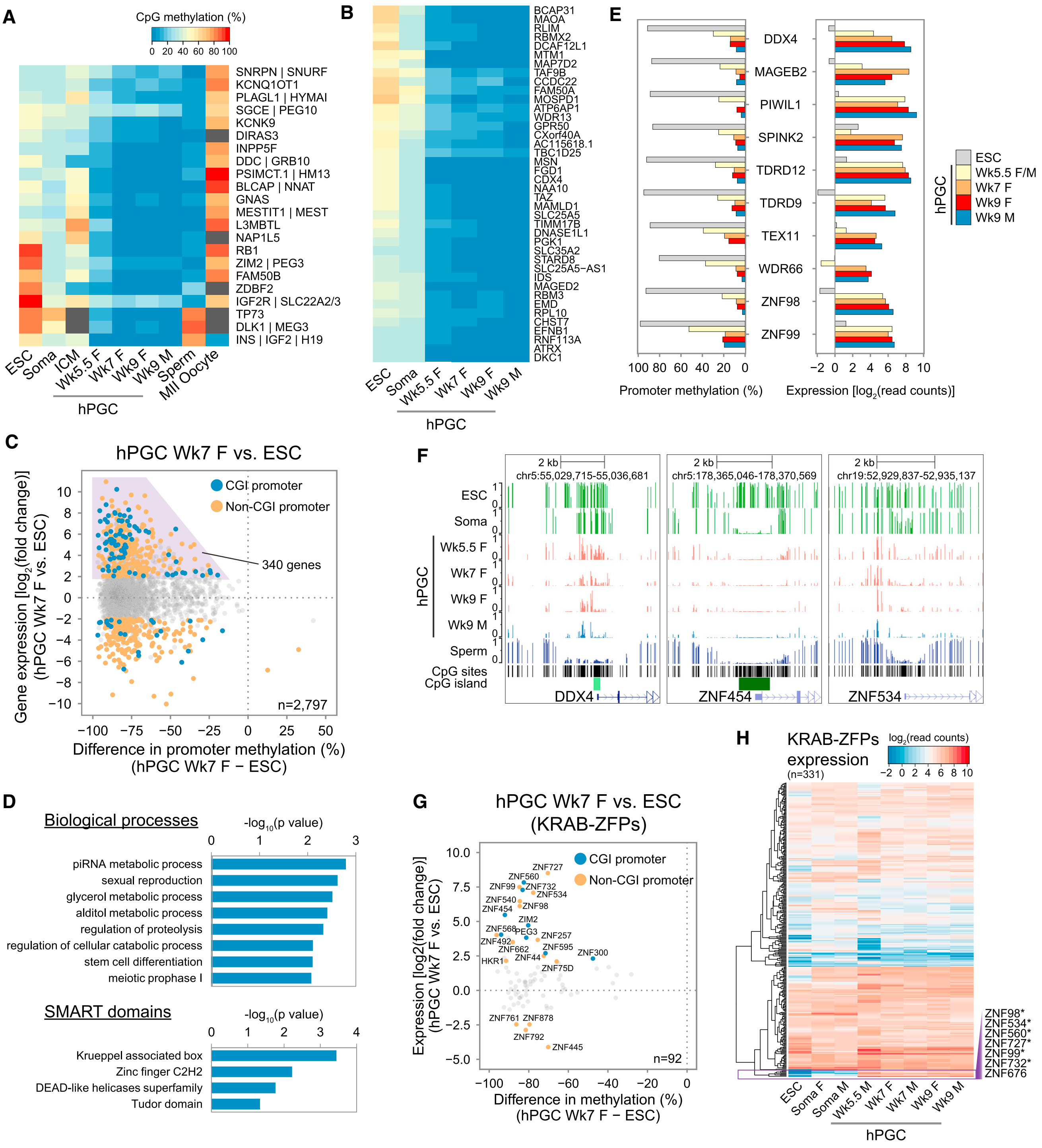

Figure 5. Imprint Erasure and Regulation of Gene Expression by Promoter Methylation

(A) DNA demethylation dynamics of ICRs in hPGCs. Published MII oocyte RRBS data are included. Gray boxes indicate ICRs that do not pass the minimum coverage thresholds.

(B) DNA demethylation dynamics of CGI-containing X chromosome promoters that are partially methylated (30%–70% methylation) in both ESCs and female gonadal somatic cells. Color key is shared with (A).

(C) Scatterplot of differential gene expression and difference in promoter methylation between Wk7 female hPGCs and ESCs. Genes with >20% promoter methylation and $\log_2$(read counts) > 3 in either samples are shown. Colored points represent differentially expressed genes ($\log_2$(fold change)>2 and $p < 0.05$). Genes upregulated in hPGCs with promoter demethylation are highlighted in the purple box.

(legend continued on next page)

CpG methylation at conserved repeat-free 1 kb tiles. Human Wk5.5 and mouse E10.5 PGCs, representing the recent arrivals into the genital ridges, showed comparable partial methylation (Figure 4E). The levels dropped to a minimum in Wk7–Wk9 hPGCs and E13.5 mPGCs. Whereas E16.5 female mPGCs remained hypomethylated, E16.5 male mPGCs had already begun de novo methylation. Consistently, unsupervised hierarchical clustering showed that the partially methylated human Wk5.5 and mouse E10.5 PGCs clustered together, whereas hypomethylated mouse and human PGCs formed another cluster, with the remethylated E16.5 male mPGCs being a distinct branch (Figure 4F). Thus, DNA demethylation dynamics of Wk5.5–Wk9 hPGCs are overall similar to E10.5–E13.5 mPGCs (Figure 1A). In contrast to male mPGCs, which remain demethylated for only a few days, male hPGCs remain hypomethylated for weeks and only showed signs of remethylation in the second trimesters (~Wk16 onward) (Wermann et al., 2010).

Imprint Erasure and X Chromosome Reactivation in hPGCs

In mPGCs, most of the ICRs retain partial methylation at 20%–50% at E10.5 (Hackett et al., 2013; Kobayashi et al., 2013). In humans, only 6 out of 22 ICRs retained >20% methylation at equivalent developmental stage (Wk5.5) (Figure 5A). These ICRs went on to become demethylated by Wk7, with the exception of *IGF2R* and *PEG10* ICRs, which seem to evade full erasure. Thus, imprint erasure in humans predominantly occurs before genital ridge colonization, which indicates earlier dynamics compared to mPGCs. This is consistent with the partial demethylation of *H19* and *GNAS* shortly after hPGCLC specification (Figure 3A).

X reactivation is another feature of PGC reprogramming (Chuva de Sousa Lopes et al., 2008; Sugimoto and Abe, 2007). DNA demethylation of the X chromosomes followed the same overall trend as that of autosomes in hPGCs (Figure S5A). As CGI promoters of the X-inactivated allele are highly methylated, we specifically looked at partially methylated X-CGI promoter (30%–70% CpG methylation) of H9 ESCs and Wk7 soma (both females). These promoters, which are potentially inactivated and methylated at the Xi, were all hypomethylated in Wk5.5–Wk9 female hPGCs (Figure 5B), confirming reactivation of the Xi as indicated by H3K27me3 immunofluorescence (Figure 3H).

Regulation of Germ Cell and KRAB-ZFP Genes by Promoter Methylation

Because most promoters were demethylated in Wk7–Wk9 hPGCs (Figure S4B), we asked whether this might cause global gene upregulation. Comparisons of gene expression and promoter methylation between hPGCs and ESCs/soma showed that >99.5% of promoters were more hypomethylated in hPGCs, with the majority (>80%) of the corresponding genes not being differentially expressed (Figure S5B). Hence, promoter demethylation was globally uncoupled with gene expression in hPGCs. Nonetheless, 340 genes (12% of total), which displayed promoter demethylation, were upregulated in hPGCs over ESCs (Figure 5C and Table S3). These genes were enriched for biological processes terms related to germ cell development (Figure 5D), such as "piRNA metabolic process" (e.g., *MAEL*, *PIWIL1*, and *PIWIL2*) and "sexual reproduction" (e.g., *NANOS3*, *HIST1H1A*, *DAZL*, and *TEX11*). They were also enriched for genes encoding protein domains such as "DEAD-like helicases" (e.g., *DDX4*, *DDX43*, and *DDX53*) and "Tudor domain" (e.g., *RNF17*, *TDRD5*, and *TDRD9*), which are involved in RNA binding and piRNA pathways. Indeed, gradual promoter demethylation, coupled with increasing expression, was observed for some of these genes in Wk5.5–Wk9 hPGCs (Figure 5E). This is reminiscent of mPGCs, where promoter demethylation activates late germ cell genes for meiosis and genome defense (Hackett et al., 2012; Maatouk et al., 2006).

Krüppel-associated box zinc finger genes (KRAB-ZFPs) constitute the largest individual family of mammalian transcriptional repressors and play pleiotropic roles, including imprint establishment, germ cell differentiation, and retrotransposon repression (Jacobs et al., 2014; Nowick et al., 2013). KRAB domain, positioned at the N terminus of DNA-binding zinc fingers, interacts with KAP-1 (also known as TRIM28), which recruits repressive complex to target sequences for heterochromatin formation and DNA methylation. Notably, some KRAB-ZFPs showed promoter demethylation and were upregulated in hPGCs over primed ESCs (Figures 5D and 5G), as is the case in soma-ESCs comparison (Figures S5C and S5D). Unsupervised hierarchical clustering of expression of all annotated KRAB-ZFPs showed a cluster of seven genes being particularly highly expressed in hPGCs (Figure 5H). Promoters of six of these KRAB-ZFPs were methylated in ESCs, but not in hPGCs, including *ZNF534*, which was also methylated and repressed in soma (Figure 5F). This implies that promoter methylation might be a general regulatory mechanism for a subset of KRAB-ZFPs in different cell types.

Demethylation and Transcriptional Dynamics of Retrotransposons

DNA methylation is important for repression of retrotransposons, which make up about half of the human genome (Burns and Boeke, 2012). We examined methylation states of major human retrotransposon classes, including long and short interspersed elements (LINEs and SINEs, respectively), SINE-variable number of tandem repeats-Alu elements (SVAs), and long terminal

(D) GO biological processes and SMART protein domain enrichment of genes highlighted in purple box of (C).
(E) Promoter methylation and expression levels of representative germ-cell-related and KRAB-ZFP genes.
(F) UCSC genome browser screenshots of CpG methylation at promoter of representative genes. Each vertical line represents one CpG site (≥5×).
(G) Scatterplot of differential gene expression and difference in promoter methylation of KRAB-ZFPs between Wk7 hPGCs and ESCs. KRAB-ZFPs with >20% promoter methylation and $\log_2$(read counts) > 3 in either samples are shown. Colored points represent differentially expressed genes.
(H) Unsupervised hierarchical clustering of KRAB-ZFPs expressions. Purple box indicates KRAB-ZFPs highly expressed in hPGCs. Asterisks indicate genes with promoter demethylation in hPGCs compared to ESCs.
See also Figure S5 and Table S3.

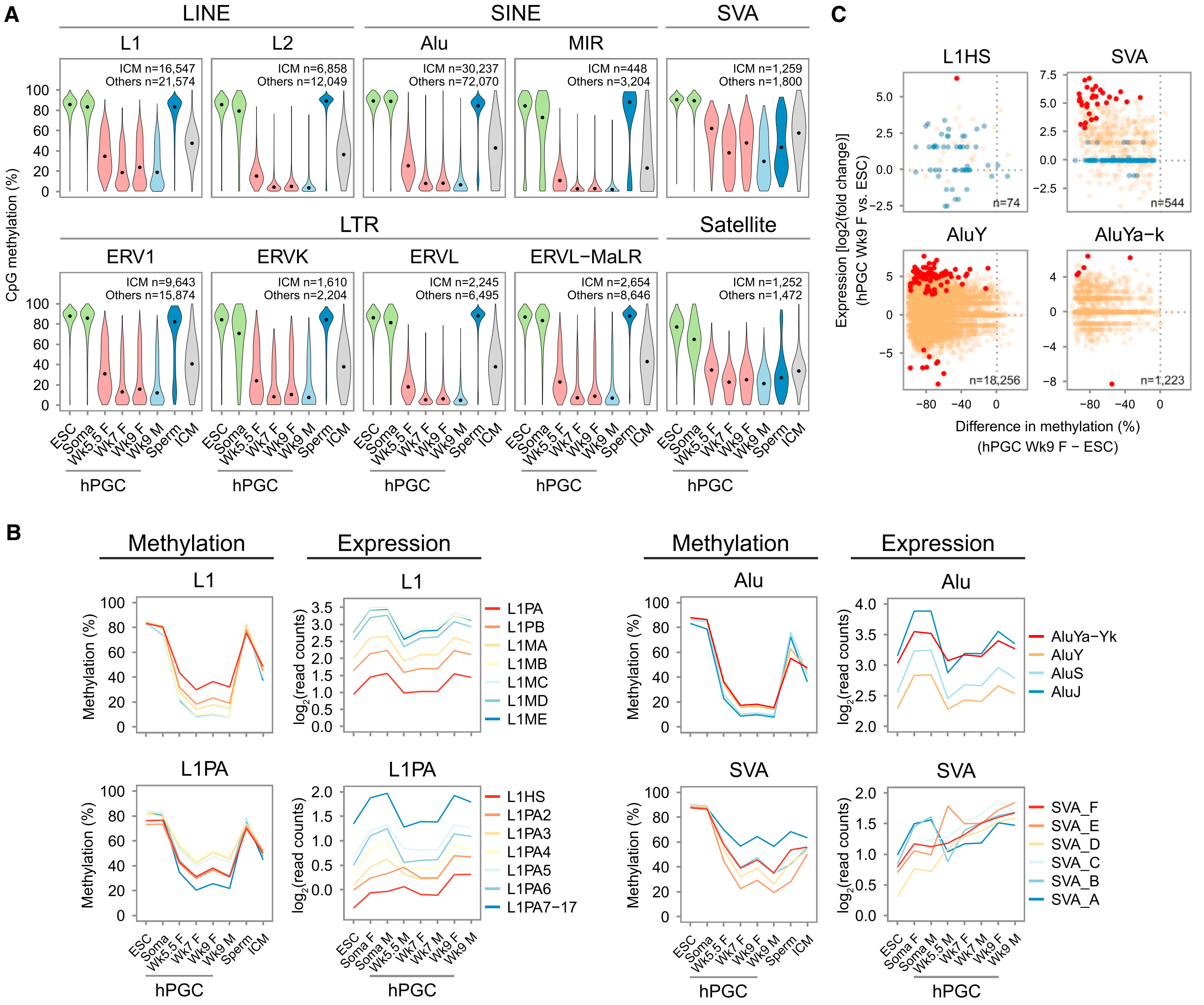

Figure 6. Demethylation and Transcriptional Dynamics of Retrotransposons

(A) Violin plots showing distribution of CpG methylation in major human repetitive elements classes and families. Common repeat loci with a minimum of 5 CpGs ($\geq 5\times$ coverage) in each sample are used. This threshold is used for all repeat methylation analyses.

(B) Average DNA methylation and expression of notable human repeat families. For each family, color keys from blue to red indicate evolutionarily older to younger retrotransposons.

(C) Scatterplot of differential expression and difference in methylation levels of potentially active retrotransposon loci between Wk9 female hPGCs and ESCs. Red points indicate differentially expressed repeat loci [$\log_2$(fold change)>2, $p < 0.05$ and $\log_2$(read counts) > 1], and blue points indicate repeat loci with low expression [$\log_2$(read counts < 0)]. Only loci that are close to full length are shown (AluY and AluYa-Yk > 268 bp; L1HS > 6,000 bp; SVA > 1,600 bp).

See also Figure S6.

repeats (LTRs). In Wk5.5–Wk9 hPGCs, the majority of retrotransposon loci underwent progressive demethylation similar to the unique portion of the genome, but a notable fraction of L1 (the younger of the two LINEs families) and SVA loci remained partially methylated (Figures 6A and S6A). In particular, half of SVA loci showed >30% methylation across all hPGC stages, and many remained partially methylated in sperm (Figure 6A) (Molaro et al., 2011), implying limited de novo methylation of partially methylated SVA loci from the first trimester to adulthood in the human male germline.

DNA demethylation of repeats in hPGCs was not accompanied by their global derepression (Figure S6B). We then focused on L1, Alu, and SVA subfamilies, which contain active members capable of retrotransposition (Hancks and Kazazian, 2012). We observed a general trend that evolutionarily younger repeat subfamilies were relatively more methylated in hPGCs than their evolutionarily older counterparts (Figure 6B). For example, L1PA, the youngest L1 subfamily, demonstrated >30% average methylation, whereas the older L1PB and L1MA-ME subfamilies were demethylated to a greater extent in chronological order of

evolutionary ages. This trend was also seen in family members of L1PA, from the younger L1PA3 to older L1PA17, except for the youngest L1HS and L1PA2 (Figure 6B), probably due to recent deletion of a KRAB-ZFP/KAP1 repressive complex target sequence (Jacobs et al., 2014). Despite partial demethylation, no prominent derepression of L1 and Alu subfamilies was observed in hPGCs compared to ESCs and soma (Figure 6B). However, hominid-specific SVA elements displayed a significant negative correlation between methylation and expression in hPGCs (Figure S6C) and were progressively upregulated in Wk5–Wk9 hPGCs upon partial demethylation (Figure 6B). Importantly, all differentially expressed and demethylated SVA loci between Wk9 hPGCs and ESCs were exclusively upregulated in hPGCs, which was not the case for other active retrotransposons (Figure 6C).

Taken together, evolutionarily youngest and currently active retrotransposons are more resistant to global demethylation. However, repeat demethylation is largely uncoupled with repeat expression (except for SVA), suggesting that additional epigenetic controls, such as repressive histone modifications (e.g., H3K9me3 and H3K9me2 in Figures 3I and 3J), might preserve hPGC genome integrity.

DNA Demethylation Escapees as Candidates for Transgenerational Epigenetic Inheritance

Next, we examined the regions that evade genome-wide DNA demethylation (referred to as "escapees") in hPGCs. We calculated significantly hyper-methylated regions in individual and pooled Wk7–Wk9 hPGC methylomes and identified a total of 116,618 escapees (median size = 1,939 bp) with $\geq$30% averaged CpG methylation. The majority (93.9%) of them had more than 10% of their genomic region covered by annotated repeats (Figure S7A). Interestingly, 7,071 escapees were predominantly depleted of retrotransposons (repeat poor, <10% overlap with repeats), out of which 1,426 were repeat free (1 kb away from any repeat subfamily). Repeat-poor escapees (median size = 1,930 bp) were located preferably at enhancers, CGI, promoters, and gene bodies (Figure 7A). Functional enrichment analysis indicated that repeat-poor escapees were frequently found in genes expressed in brain (Figure 7B) and participated in neural development (Figure S7C). Comparison of the escapee genes with the NHGRI GWAS catalog revealed characteristic trait and disease associations, such as "obesity-related traits," "schizophrenia," and "multiple sclerosis" (Figure 7C). Notably, some escapees displayed variations in methylation levels among the four most hypomethylated individual embryos in our study (Figures S7D and S7E). It will be of interest to examine these escapee regions on a larger scale.

We assessed the epigenetic landscapes of resistant loci and found that H3K9me3 primarily marked both repeat-rich and repeat-poor escapees in selected in vivo cell types (see Supplemental Experimental Procedures) (Figure 7D). Resistant regions were also enriched for KAP1 binding sites of ESCs (Figure S7F), suggesting that they might be targeted by KRAB-ZFP/KAP1 repressive complex that induces heterochromatin formation. ZFP57 (or also called ZNF698) is a notable KRAB-ZFP protein known to establish/maintain methylation of ICRs through recognition of consensus methylated DNA sequence (Quenneville et al., 2011). We tested for enrichment of a predicted human ZFP57 motif in escapees. Strong enrichment was found for repeat-rich escapees associated with SVA and L1PA retroelements, whereas enrichment for repeat-poor escapees was moderate (Figure 7E). Analysis for ZNF98, a hPGC-enriched KRAB-ZFP with a motif distinct to ZFP57 (Figure 5H), showed enrichment for L1PAs and LTRs but depletion of repeat-free regions that lacked consensus sequence (Figure S7H). Thus, the plethora of KRAB-ZFPs expressed in hPGCs might confer demethylation resistance in various retrotransposon families with different consensus sequences (Jacobs et al., 2014). Intriguingly, repeat-poor escapees also included KRAB-ZFPs themselves (Figure S7G), implying potential auto-regulatory mechanism to retain methylation within KRAB-ZFP gene bodies.

Making use of a recent high-coverage BS-seq dataset (Okae et al., 2014), we traced the fate of escapees in gametes and preimplantation embryos (Figures 7F and S7I). In particular, we clustered the common repeat-poor escapees of Wk7–9 hPGCs, together with gametes and blastocyst (Figure 7F). Most escapees were partially methylated in ICM (cluster 2–5), indicating that hPGC escapees were generally not erased during both waves of epigenetic reprogramming. The majority of escapees became highly methylated in both sperm and oocytes (clusters 3 and 5) and were therefore fully "programmed" during later germ cell development. Notably, some escapees were methylated in either sperm (cluster 4) or oocytes (cluster 2) and demonstrated partial methylation in gametes of the opposite gender. These groups were partially programmed at all stages of germline methylation cycle, and thus, variations in methylation levels might contribute to transgenerational epigenetic inheritance.

A comparable number of regions that are resistant to DNA demethylation and do not intersect with methylated IAP repeats was previously reported in mPGCs (Hackett et al., 2013; Seisenberger et al., 2012). Human and mouse escapees were generally not well conserved at sequence level. Out of 3,246 conserved regions with human escapees, only 104 (3%) exhibited more than 10% average methylation in the orthologous region on mouse genome at the most demethylated stage (E13.5). The few sequence-conserved escapees were frequently found in gene bodies (e.g., SRRM2 in Figure 7G). We thus compiled all hyper-methylated regions of E13.5 mPGCs in the mouse genome and compared escapee conservation in homologous gene level. Out of 2,742 genes detected in human and 3,552 genes detected in mouse with repeat-poor escapees in their gene bodies (>30% methylation), 794 of them were in common (p value = 8.75×10^{-75}) (Figure 7H). This conserved set of genes in human and mice was functionally biased toward brain and growth-related functions. For example, *TACC2*, an androgen-responsive cell cycle regulator, exhibited an escapee region across its promoter in humans, which was not sequence-conserved in mice (Figure S7B). However, a differentially methylated region was detected in the gene body of the homologous *Tacc2* gene in sperm of mice that were undernourished in utero, and its potential intergenerational phenotypic impact was reported recently (Radford et al., 2014). These escapees might therefore be important in the perspective of transgenerational epigenetic inheritance.

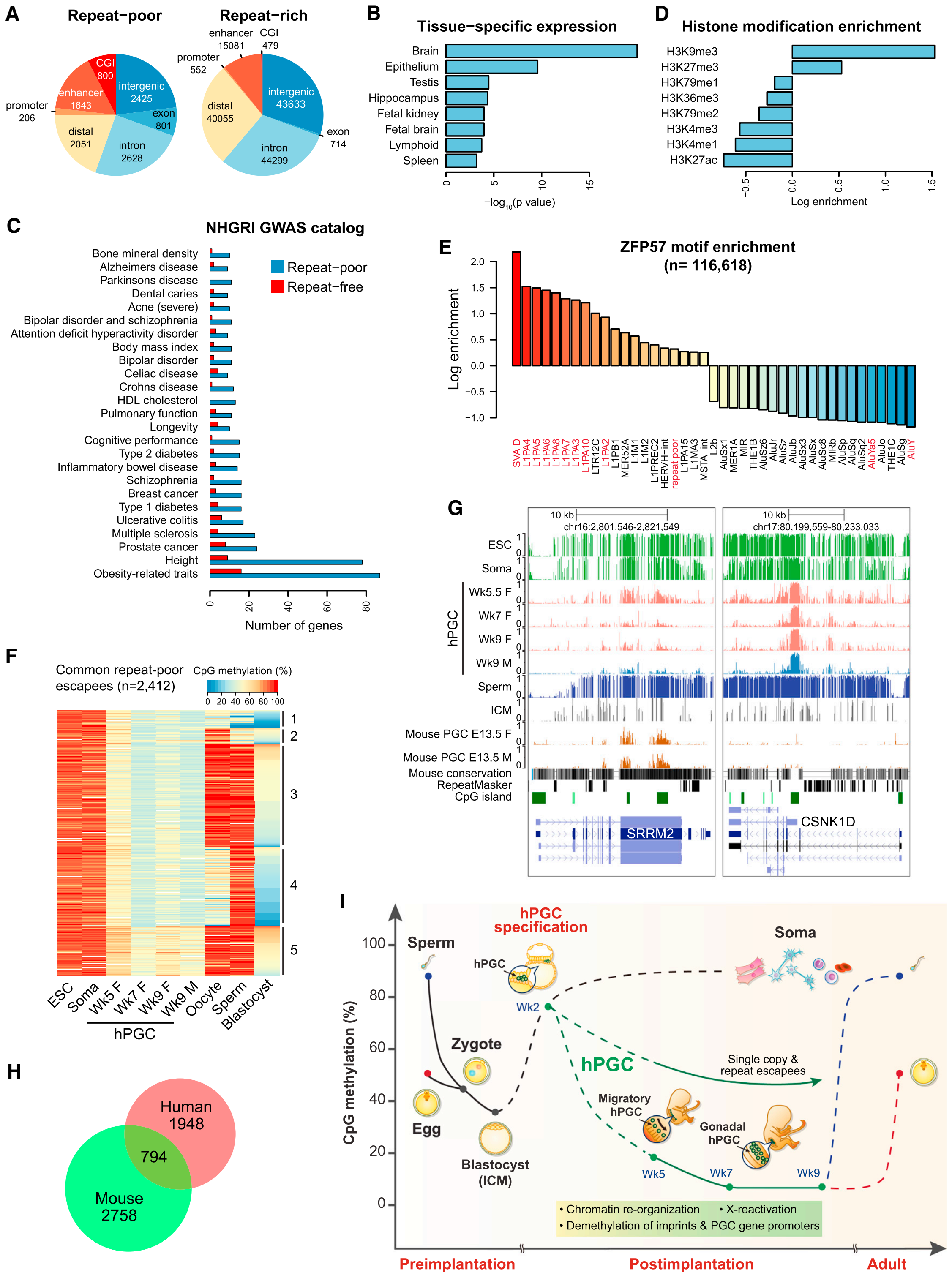

Figure 7. DNA Demethylation Escapees as Candidates for Transgenerational Epigenetic Inheritance

(A) Genomic feature distribution of repeat-poor (<10% overlap with repeats) and repeat-rich (≥10% overlap) escapees.

(B) Enrichment analysis of tissue-specific expression (UniProt UP_TISSUE) of 2,092 protein-coding genes with escapees in their gene bodies.

(C) Human diseases and traits associated with genes with repeat-poor and repeat-free escapees in their gene bodies.

(D) Enrichment of epigenetic modifications in repeat-poor escapees.

(legend continued on next page)

DISCUSSION

Through systematic investigations on nascent hPGCLCs and embryonic hPGCs, we provide comprehensive insight on the human germline at a critical juncture of extensive epigenetic reprogramming (Figure 7I). hPGCs exhibit expression of naive pluripotency genes, KLF4 and TFCP2L1, as well as some lineage specifiers, such as TEAD4. This unique gene regulatory network established by SOX17 and BLIMP1 (Irie et al., 2015) initiates and maintains the human germline epigenetic program. In particular, hPGCs undergo chromatin reorganization and comprehensive DNA demethylation, which entails 5mC erasure at imprints, transposable elements, and promoters of methylation-sensitive germline and KRAB-ZFP genes. Despite global hypomethylation, we found regions that are variably resistant to DNA demethylation, which are potential mediators of transgenerational epigenetic inheritance.

The gene regulatory network for hPGC specification and maintenance is distinct from that in mice. During mPGC specification, BLIMP1 and PRDM14, together with TFAP2C, upregulate germ cell and pluripotency genes, repress somatic fates, and initiate epigenetic reprogramming (Magnúsdóttir et al., 2013; Nakaki et al., 2013). While we showed that BLIMP1 plays a similar role in the human germline, it is striking that SOX17 is upstream of BLIMP1 during human germ cell specification (Irie et al., 2015). Loss of SOX17 abolishes hPGCLC specification, including the expression of BLIMP1 and other germ cell genes, whereas SOX17 overexpression alone reinstates the hPGC transcriptional program. Notably, PRDM14 is upregulated later than other germ cell genes (Irie et al., 2015), and its expression in hPGCLCs and hPGCs is >4 times lower than that in ESCs. PRDM14 is apparently downstream of BLIMP1 during hPGCLC specification (Figure 2C). While a recent report claimed that reduced levels of *PRDM14* (knockdown with 60%–70% efficiency) did not affect hPGCLC specification (Sugawa et al., 2015), knockout studies are warranted to determine its role in the human germline.

Preimplantation embryos (Guo et al., 2014; Smith et al., 2014), ground state human ESCs (Takashima et al., 2014), and hPGCs share some features of genome-wide epigenetic reprogramming, including global DNA demethylation, chromatin reorganization, and potentially X reactivation. Interestingly, they also share expression of some pluripotency genes, including TFCP2L1, KLF4, NANOG, and OCT4. It is possible that these factors may contribute to epigenome resetting and global hypomethylation. However, the extent of DNA demethylation is far more comprehensive in hPGCs than seen in other instances. Following initiation of DNA demethylation in nascent hPGCLCs, the process continues in Wk4–Wk9 in vivo hPGCs toward comprehensive erasure. The germline gene regulatory network, consisting of BLIMP1 and SOX17 among other factors, likely constitutes a unique "reset switch" that initiates and sustains robust repression of DNA methylation pathways and activation of TET-mediated hydroxymethylation.

Global DNA demethylation occurs within ~5 days in mPGCs, but this takes ~4 weeks in humans (Figure 1A) and is also protracted in porcine PGCs (Hyldig et al., 2011). While mPGCs have an ~12 hr cell cycle, the doubling time of hPGCs approximates to ~6 days (Bendsen et al., 2006). The protracted DNA demethylation of hPGCs is consistent with replication-coupled dilution of 5mC and 5hmC. Interestingly, nascent hPGCLCs already demonstrate 5mC reduction with enrichment of 5hmC at imprints and at promoters of germ cell genes. In vivo hPGCs also show earlier loss of DNA methylation at these loci compared to mice (Hackett et al., 2013; Yamaguchi et al., 2013), suggesting earlier TET-mediated demethylation dynamics in humans.

DNA methylation appears to be the primary mechanism to repress SVAs, the hominid-specific and the youngest retrotransposon family in the primate order. Because SVAs remain partially methylated in sperm, there may be a prolonged period of SVA expression and transposition. SVAs mobilization is, however, dependent on L1HS, which encodes RNA-binding chaperone ORF1p and endonuclease and reverse-transcriptase-containing ORF2p (Hancks and Kazazian, 2012). L1HS remains repressed in hPGCs (Figure 5C), possibly by the PIWI-piRNA pathway (Pezic et al., 2014), as the key components (e.g., PIWIL1, PIWIL2 and PIWL4) are upregulated in hPGCs (Figure 2D). This would therefore limit retrotranspositions of SVA and other elements in the germline.

The comprehensive erasure of epigenetic modifications in the preimplantation embryo and the germline (Figure 7I) is apparently the major barrier to epigenetic inheritance in mammals (Heard and Martienssen, 2014). However, the detection of DNA methylation-resistant escapees in mouse (Hackett et al., 2013; Seisenberger et al., 2012) and now in the human germline is noteworthy. Some escapees were incompletely reprogrammed in hPGCs, gametes, and preimplantation embryos. These regions may be sensitive to environmentally induced variations in methylation in individual embryo that could persist over a short term or might even become heritable. As such, they are potential candidates for transgenerational epigenetic inheritance. Notably, many of the escapee-associated genes are expressed in brain, with potential links to neurological and metabolic disorders in humans. It is possible that the purpose of such heritable epigenetic modifications might normally be beneficial by allowing more flexible and environmentally responsive phenotypic

(E) Enrichment of ZFP57 motif among repeat-poor escapees and methylated retrotransposons (with >700 copies).

(F) K-means clustering of DNA methylation levels of Wk7-9 hPGCs common repeat-poor escapees across germline development.

(G) UCSC genome browser screenshots of two representative genes with escapees (repeat free). Escapee region of *SRRM2* is conserved between human and mouse, whereas that of *CSNK1D* overlaps with CGI at an alternative promoter.

(H) Venn diagram showing overlap of homologous genes with repeat-free escapees in their gene bodies between human and mouse.

(I) Schematic showing dynamics of preimplantation and germline epigenetic reprogramming in humans. hPGCs undergo the most comprehensive wave of global DNA demethylation, which reaches a minimum of ~5% CpG methylation at weeks 7–9. Some single copy and repeat loci remain methylated and are candidates for transgenerational epigenetic inheritance. Dotted line indicates postulated methylation dynamics.

See also Figure S7.

diversity than is possible with genetic information. A significant fraction of escapees overlaps with gene bodies and regulatory regions, including enhancers, promoters, and CGIs. Methylation at alternative promoters or splice sites can affect transcript variants expression, whereas enhancer methylation may modulate gene expression (Jones, 2012). These are potential mechanisms for translating DNA methylation information to phenotypes. Overall, the detection of escapees represents an important new avenue for investigation on epigenetic inheritance in humans.

What is the underlying mechanism(s) for demethylation resistance in a globally hypomethylated environment? We observed that some escapees are enriched for H3K9me3, KAP1, and ZFP57 motif, suggesting a role for KRAB-ZFP/KAP1 complex in conferring DNA methylation by recruitment of residual DNA methylation machineries. In particular, hPGCs show a plethora of highly expressed KRAB-ZFPs genes, with a subset being regulated by promoter methylation. This constitutes a diverse pool of sequence-specific DNA binding factors, which could potentially confer and/or maintain DNA methylation at escapee loci with target motifs. In mPGCs, CGI in the neighborhood of an IAP repeat are more resistant to demethylation (Seisenberger et al., 2012). Methylated repeat families, such as SVAs and L1PAs, may have a similar effect on neighboring regions in humans. Interestingly, as escapees are targeted for hydroxymethylation in mPGCs (Hackett et al., 2013), it is possible that these loci undergo a process of reiterative de novo methylation and hydroxymethylation.

In conclusion, we observed a unique transcriptional state associated with hPGCs, which provides a context for comprehensive germline epigenetic reprogramming. Our in vitro model for the specification of hPGCLCs from ESCs and iPSCs (Irie et al., 2015) now offers an opportunity to functionally test some of these new findings. The combination of in vivo and in vitro approaches will provide comprehensive insights on human germline biology and inheritance.

EXPERIMENTAL PROCEDURES

Collection of Human Embryonic Samples

Human embryonic tissues were collected from medical or surgical terminated embryos at Addenbrooke's Hospital (Cambridge, UK) under permission from NHS Research Ethical Committee (96/085). Developmental stage of human embryo was determined by crown-rump length and anatomical features (e.g., limbs and digits morphology) with reference to Carnegie staging. Gender was determined by sex determination PCR as described (Irie et al., 2015). To isolate hPGCs, genital ridges were dissociated by TrypLE Express and resuspended in FACS medium with Alexa Fluor 488-anti-alkaline phosphatase and PerCP-Cy5.5-anti-CD117 antibodies (BD PharMingen). After incubation, cells were pelleted, resuspended and sorted with S3 Cell Sorter (Bio-Rad). For immunofluorescence, embryos or genital ridges were fixed in 4% formaldehyde and prepared as cryosections as described (Irie et al., 2015).

RNA-Seq Analysis

Total RNA was extracted by PicoPure RNA Isolation Kit (Applied Biosystems) and converted into cDNA by Ovation RNA-Seq System V2 (Nugen). Amplified cDNA was then sonicated into ~250 bp by Covaris S220. Multiplexed RNA-seq libraries were constructed by Ovation Rapid DR Multiplex System (Nugen) and subjected to single-end 50 bp sequencing on HiSeq 2500 (Illumina). Adaptor-and quality-trimmed RNA-seq reads were mapped to the human reference genomes (UCSC GRCh37/hg19) using *TopHat2* guided by ENSEMBL 74 gene models. Raw counts per transcripts were obtained using *featureCounts*. Normalization and differential expression analysis were performed by *DESeq*.

BS-Seq Analysis

PBAT libraries were constructed as described by Kobayashi et al. (2013) and subjected to single/paired-end 100 bp sequencing on HiSeq 2500 (Illumina). Adaptor- and quality-trimmed reads were mapped to in silico bisulfite-converted human reference genome (GRCh37/hg19) by *Bismark*. Average methylation levels for each CpG sites and annotated genomic regions were called with *MethPipe*.

Detailed experimental methods and bioinformatics analysis are available in the Supplemental Experimental Procedures.

ACCESSION NUMBERS

The accession number for the RNA-seq and BS-seq data reported in this paper is NCBI SRA: SRP057098.

SUPPLEMENTAL INFORMATION

Supplemental Information includes Supplemental Experimental Procedures, seven figures, and four tables and can be found with this article online at http://dx.doi.org/10.1016/j.cell.2015.04.053.

AUTHOR CONTRIBUTIONS

W.W.C.T. and M.A.S. conceived the project and designed experiments; W.W.C.T. collected human materials and performed experiments with help from N.I., H.G.L., and J.A.H.; S.D. carried out bioinformatics analysis with W.W.C.T. and C.R.B.; V.I.F and P.F.C. advised on isolation of hPGCs; W.W.C.T., S.D., J.A.H., and M.A.S. wrote the manuscript.

ACKNOWLEDGMENTS

We thank Roger Barker, Xiaoling He, and Pam Tyers for help with collection of human embryos and Andy Riddell for cell sorting. We acknowledge Nils Grabole and Roopsha Sengupta for technical advice, Toshihiro Kobayashi for making schematics, and all members of the M.A.S. lab for discussions. W.W.C.T is supported by Croucher Foundation and Cambridge Trust. N.I. is supported by BIRAX. P.F.C. is a Wellcome Trust Senior Fellow in Clinical Science (101876/Z/13/Z) and a UK NIHR Senior Investigator with additional support from the Wellcome Trust Centre for Mitochondrial Research (096919Z/11/Z). M.A.S. is supported by HFSP and a Wellcome Trust Investigator Award.

Received: January 28, 2015
Revised: March 27, 2015
Accepted: April 14, 2015
Published: June 4, 2015

REFERENCES

Bendsen, E., Byskov, A.G., Andersen, C.Y., and Westergaard, L.G. (2006). Number of germ cells and somatic cells in human fetal ovaries during the first weeks after sex differentiation. Hum. Reprod. *21*, 30–35.

Burns, K.H., and Boeke, J.D. (2012). Human transposon tectonics. Cell *149*, 740–752.

Chuva de Sousa Lopes, S.M., Hayashi, K., Shovlin, T.C., Mifsud, W., Surani, M.A., and McLaren, A. (2008). X chromosome activity in mouse XX primordial germ cells. PLoS Genet. *4*, e30.

Dawlaty, M.M., Breiling, A., Le, T., Raddatz, G., Barrasa, M.I., Cheng, A.W., Gao, Q., Powell, B.E., Li, Z., Xu, M., et al. (2013). Combined deficiency of Tet1 and Tet2 causes epigenetic abnormalities but is compatible with postnatal development. Dev. Cell *24*, 310–323.

Gkountela, S., Li, Z., Vincent, J.J., Zhang, K.X., Chen, A., Pellegrini, M., and Clark, A.T. (2013). The ontogeny of cKIT+ human primordial germ cells proves

to be a resource for human germ line reprogramming, imprint erasure and in vitro differentiation. Nat. Cell Biol. *15*, 113–122.

Guibert, S., Forné, T., and Weber, M. (2012). Global profiling of DNA methylation erasure in mouse primordial germ cells. Genome Res. *22*, 633–641.

Guo, H., Zhu, P., Yan, L., Li, R., Hu, B., Lian, Y., Yan, J., Ren, X., Lin, S., Li, J., et al. (2014). The DNA methylation landscape of human early embryos. Nature *511*, 606–610.

Hackett, J.A., Reddington, J.P., Nestor, C.E., Dunican, D.S., Branco, M.R., Reichmann, J., Reik, W., Surani, M.A., Adams, I.R., and Meehan, R.R. (2012). Promoter DNA methylation couples genome-defence mechanisms to epigenetic reprogramming in the mouse germline. Development *139*, 3623–3632.

Hackett, J.A., Sengupta, R., Zylicz, J.J., Murakami, K., Lee, C., Down, T.A., and Surani, M.A. (2013). Germline DNA demethylation dynamics and imprint erasure through 5-hydroxymethylcytosine. Science *339*, 448–452.

Hajkova, P., Erhardt, S., Lane, N., Haaf, T., El-Maarri, O., Reik, W., Walter, J., and Surani, M.A. (2002). Epigenetic reprogramming in mouse primordial germ cells. Mech. Dev. *117*, 15–23.

Hancks, D.C., and Kazazian, H.H., Jr. (2012). Active human retrotransposons: variation and disease. Curr. Opin. Genet. Dev. *22*, 191–203.

Heard, E., and Martienssen, R.A. (2014). Transgenerational epigenetic inheritance: myths and mechanisms. Cell *157*, 95–109.

Hyldig, S.M., Croxall, N., Contreras, D.A., Thomsen, P.D., and Alberio, R. (2011). Epigenetic reprogramming in the porcine germ line. BMC Dev. Biol. *11*, 11.

Irie, N., Weinberger, L., Tang, W.W., Kobayashi, T., Viukov, S., Manor, Y.S., Dietmann, S., Hanna, J.H., and Surani, M.A. (2015). SOX17 is a critical specifier of human primordial germ cell fate. Cell *160*, 253–268.

Jacobs, F.M., Greenberg, D., Nguyen, N., Haeussler, M., Ewing, A.D., Katzman, S., Paten, B., Salama, S.R., and Haussler, D. (2014). An evolutionary arms race between KRAB zinc-finger genes ZNF91/93 and SVA/L1 retrotransposons. Nature *516*, 242–245.

Jones, P.A. (2012). Functions of DNA methylation: islands, start sites, gene bodies and beyond. Nat. Rev. Genet. *13*, 484–492.

Kagiwada, S., Kurimoto, K., Hirota, T., Yamaji, M., and Saitou, M. (2013). Replication-coupled passive DNA demethylation for the erasure of genome imprints in mice. EMBO J. *32*, 340–353.

Kobayashi, H., Sakurai, T., Miura, F., Imai, M., Mochiduki, K., Yanagisawa, E., Sakashita, A., Wakai, T., Suzuki, Y., Ito, T., et al. (2013). High-resolution DNA methylome analysis of primordial germ cells identifies gender-specific reprogramming in mice. Genome Res. *23*, 616–627.

Leitch, H.G., Tang, W.W., and Surani, M.A. (2013). Primordial germ-cell development and epigenetic reprogramming in mammals. Curr. Top. Dev. Biol. *104*, 149–187.

Lu, X., Sachs, F., Ramsay, L., Jacques, P.E., Göke, J., Bourque, G., and Ng, H.H. (2014). The retrovirus HERVH is a long noncoding RNA required for human embryonic stem cell identity. Nat. Struct. Mol. Biol. *21*, 423–425.

Maatouk, D.M., Kellam, L.D., Mann, M.R., Lei, H., Li, E., Bartolomei, M.S., and Resnick, J.L. (2006). DNA methylation is a primary mechanism for silencing postmigratory primordial germ cell genes in both germ cell and somatic cell lineages. Development *133*, 3411–3418.

Magnúsdóttir, E., Dietmann, S., Murakami, K., Günesdogan, U., Tang, F., Bao, S., Diamanti, E., Lao, K., Gottgens, B., and Azim Surani, M. (2013). A tripartite transcription factor network regulates primordial germ cell specification in mice. Nat. Cell Biol. *15*, 905–915.

Miura, F., Enomoto, Y., Dairiki, R., and Ito, T. (2012). Amplification-free whole-genome bisulfite sequencing by post-bisulfite adaptor tagging. Nucleic Acids Res. *40*, e136.

Molaro, A., Hodges, E., Fang, F., Song, Q., McCombie, W.R., Hannon, G.J., and Smith, A.D. (2011). Sperm methylation profiles reveal features of epigenetic inheritance and evolution in primates. Cell *146*, 1029–1041.

Nakaki, F., Hayashi, K., Ohta, H., Kurimoto, K., Yabuta, Y., and Saitou, M. (2013). Induction of mouse germ-cell fate by transcription factors in vitro. Nature *501*, 222–226.

Nowick, K., Carneiro, M., and Faria, R. (2013). A prominent role of KRAB-ZNF transcription factors in mammalian speciation? Trends Genet. *29*, 130–139.

Okae, H., Chiba, H., Hiura, H., Hamada, H., Sato, A., Utsunomiya, T., Kikuchi, H., Yoshida, H., Tanaka, A., Suyama, M., and Arima, T. (2014). Genome-wide analysis of DNA methylation dynamics during early human development. PLoS Genet. *10*, e1004868.

Pezic, D., Manakov, S.A., Sachidanandam, R., and Aravin, A.A. (2014). piRNA pathway targets active LINE1 elements to establish the repressive H3K9me3 mark in germ cells. Genes Dev. *28*, 1410–1428.

Quenneville, S., Verde, G., Corsinotti, A., Kapopoulou, A., Jakobsson, J., Offner, S., Baglivo, I., Pedone, P.V., Grimaldi, G., Riccio, A., and Trono, D. (2011). In embryonic stem cells, ZFP57/KAP1 recognize a methylated hexanucleotide to affect chromatin and DNA methylation of imprinting control regions. Mol. Cell *44*, 361–372.

Radford, E.J., Ito, M., Shi, H., Corish, J.A., Yamazawa, K., Isganaitis, E., Seisenberger, S., Hore, T.A., Reik, W., Erkek, S., et al. (2014). In utero effects. In utero undernourishment perturbs the adult sperm methylome and intergenerational metabolism. Science *345*, 1255903.

Sasaki, H. (2010). Mechanisms of trophectoderm fate specification in preimplantation mouse development. Dev. Growth Differ. *52*, 263–273.

Seisenberger, S., Andrews, S., Krueger, F., Arand, J., Walter, J., Santos, F., Popp, C., Thienpont, B., Dean, W., and Reik, W. (2012). The dynamics of genome-wide DNA methylation reprogramming in mouse primordial germ cells. Mol. Cell *48*, 849–862.

Seki, Y., Hayashi, K., Itoh, K., Mizugaki, M., Saitou, M., and Matsui, Y. (2005). Extensive and orderly reprogramming of genome-wide chromatin modifications associated with specification and early development of germ cells in mice. Dev. Biol. *278*, 440–458.

Smith, Z.D., Chan, M.M., Humm, K.C., Karnik, R., Mekhoubad, S., Regev, A., Eggan, K., and Meissner, A. (2014). DNA methylation dynamics of the human preimplantation embryo. Nature *511*, 611–615.

Sugawa, F., Araúzo-Bravo, M.J., Yoon, J., Kim, K.P., Aramaki, S., Wu, G., Stehling, M., Psathaki, O.E., Hübner, K., and Schöler, H.R. (2015). Human primordial germ cell commitment in vitro associates with a unique PRDM14 expression profile. EMBO J. *34*, 1009–1024.

Sugimoto, M., and Abe, K. (2007). X chromosome reactivation initiates in nascent primordial germ cells in mice. PLoS Genet. *3*, e116.

Takashima, Y., Guo, G., Loos, R., Nichols, J., Ficz, G., Krueger, F., Oxley, D., Santos, F., Clarke, J., Mansfield, W., et al. (2014). Resetting transcription factor control circuitry toward ground-state pluripotency in human. Cell *158*, 1254–1269.

Wermann, H., Stoop, H., Gillis, A.J., Honecker, F., van Gurp, R.J., Ammerpohl, O., Richter, J., Oosterhuis, J.W., Bokemeyer, C., and Looijenga, L.H. (2010). Global DNA methylation in fetal human germ cells and germ cell tumours: association with differentiation and cisplatin resistance. J. Pathol. *221*, 433–442.

Yamaguchi, S., Hong, K., Liu, R., Inoue, A., Shen, L., Zhang, K., and Zhang, Y. (2013). Dynamics of 5-methylcytosine and 5-hydroxymethylcytosine during germ cell reprogramming. Cell Res. *23*, 329–339.

Cell

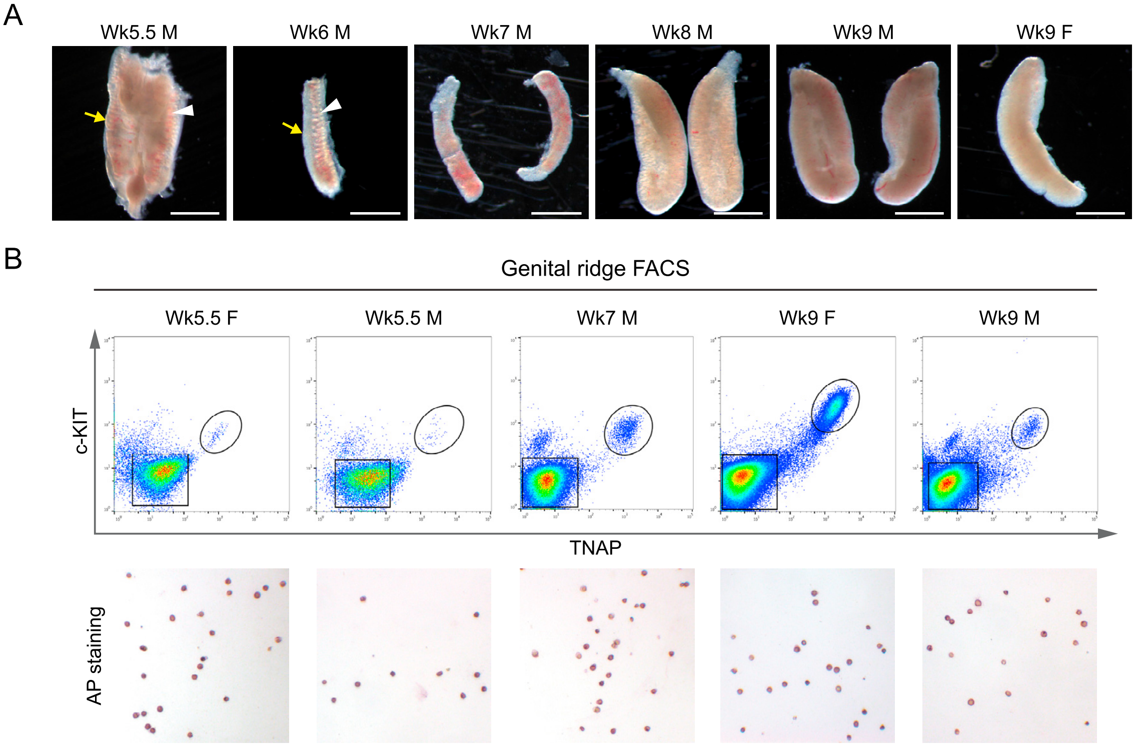

C

Embryos samples

Embryo identifier	Developmental week (Carnegie stage)	Gender	Sample	Number of cells	
				RNA-Seq	BS-Seq
1	Wk5.5 (CS15)	F	hPGC	-	142
2	Wk5.5 (CS16)	F	hPGC	-	372
3	Wk5.5 (CS16)	M	hPGC	99	-
4	Wk7 (CS19)	F	hPGC	1000	1000
			Soma	5000	5000
5	Wk7 (CS20)	F	hPGC	1000	2221
			Soma	3000	-
6	Wk7 (CS19)	F	hPGC	1689	-
			Soma	10000	-
7	Wk7 (CS19)	M	hPGC	1045*	-
8	Wk7 (CS19)	M	Soma	10000*	-
9	Wk7 (CS19)	M	hPGC	1500*	-
			Soma	10000*	-
10	Wk9 (>CS23)	F	hPGC	1000	5000
11	Wk9 (>CS23)	F	hPGC	-	2642
12	Wk9 (>CS23)	F	hPGC	2284	-
13	Wk9 (>CS23)	M	hPGC	2123	3026
14	Wk9 (>CS23)	M	hPGC	566	1704

In vitro samples

Descriptions	Gender	Sample	Number of cells	
			RNA-Seq	BS-Seq
Conventional H9 ESC	F	ESC #1	1000	1000
		ESC #2	1000	1000
Wild-type day 4 hPGCLC	M	hPGCLC WT #1	1170*	-
		hPGCLC WT #2	570*	-
BLIMP1 mutant day 4 hPGCLC	M	hPGCLC mutant #1	10000	-
		hPGCLC mutant #1	3000	-

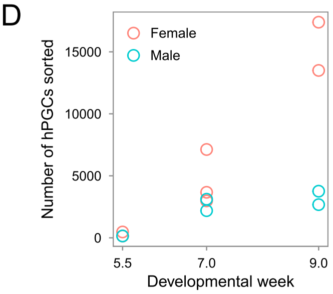

Figure S1. Isolation of hPGCs by FACS for RNA-Seq and BS-Seq, Related to Figure 1

(A) Morphology of Wk5.5-Wk9 human genital ridges. The ridge first appears as a thin coelomic epithelium (yellow arrow) adjacent to the mesonephros (white arrowhead) at Wk5.5 and subsequently develops into distinct genital ridges (Wk6-9). Sexual differentiation begins at Wk6 and the morphological differences of male (M) and female (F) ridges become apparent at Wk8-9. Scale bars, 1 mm.

(B) Isolation of hPGCs from Wk5.5 to Wk9 genital ridges by FACS with cell surface markers TNAP and c-KIT. TNAP-high and c-KIT-high population was collected for alkaline phosphatase staining and consistently showed > 97% of AP positive hPGCs (red).

(C) Details of in vivo and in vitro samples collected for RNA-Seq and BS-seq. Asterisks indicate RNA-Seq samples published in a previous study (Irie et al., 2015).

(D) Number of hPGCs sorted per embryo at Wk5.5 to Wk9.

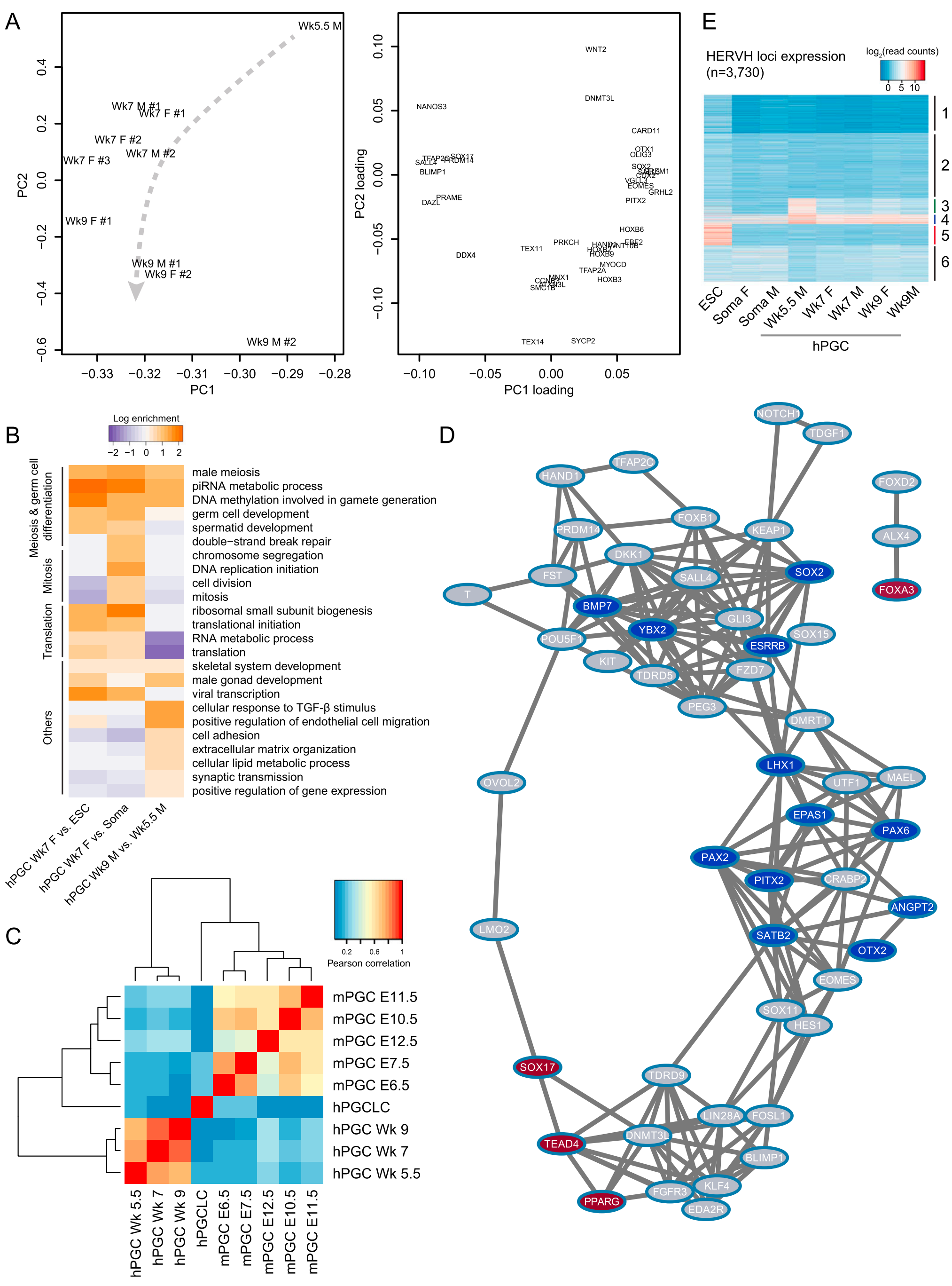

Figure S2. Transcriptional Dynamics of Human and Mouse PGCs, Related to Figure 2

(A) Two-dimensional PCA plot (PC2 against PC1) and gene loading plot. Arrow line indicates developmental progression of hPGCs from Wk5.5 to Wk9.

(B) Gene ontology biological processes enrichment heatmap of upregulated genes [log_2(fold change)>2, $p < 0.05$] in hPGCs in comparison to indicated samples.

(C) Correlation of transcription profiles of human and mouse PGCs of various stages. The comparison included hPGCs, hPGCLCs (Irie et al., 2015) and mPGCs of various stages (Magnúsdóttir et al., 2013). Only genes expressed in both human and mouse cells (log_2(read counts) > 3) but absent in soma were used for analysis. Expression levels were averaged over biological replicates.

(D) Co-expression network analysis of human and mouse PGCs. Grey indicates co-expressed genes; red indicates genes upregulated in humans; blue indicates genes upregulated in mice.

(E) K-means clustering of individual HERVH loci RNA expression into 6 dynamics.

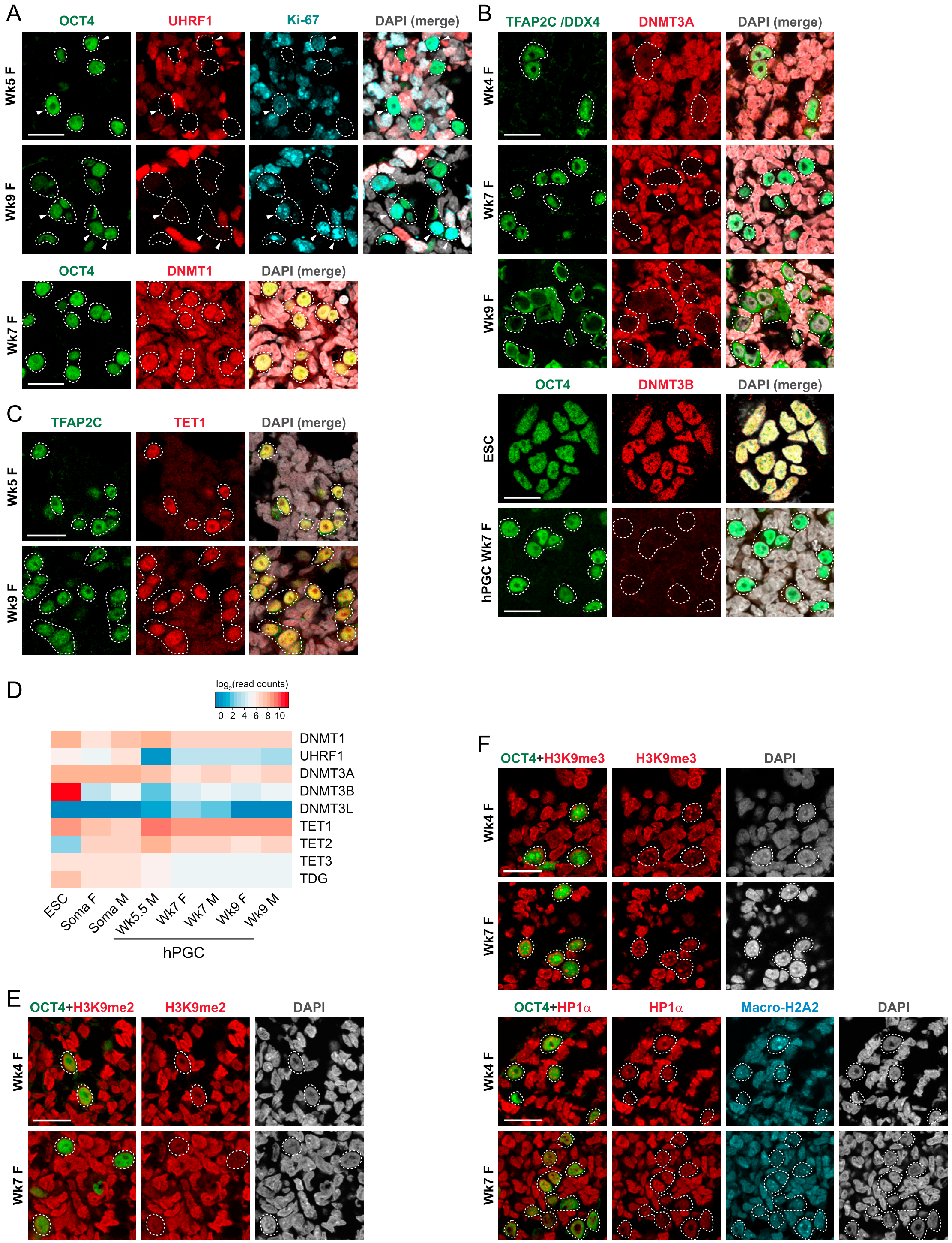

Figure S3. Global Epigenetic Dynamics in hPGCs, Related to Figure 3

(A–C) Immunofluorescence analysis for (A) UHRF1 and DNMT1; (B) DNMT3A and DNMT3B and (C) TET1 on human embryo cryosections. Arrowheads in (A) indicate examples of Ki-67-positive (proliferating) hPGCs which are UHRF1-negative. Conventional ESCs [4th row in (B)], which strongly expresses DNMT3B, is used as a positive control in contrast to the absence of DNMT3B signals in Wk7 F ridges. Scale bars, 20 μm.

(D) Expression of epigenetic modifiers in various human samples by RNA-Seq.

(E and F) Immunofluorescence analysis for (E) H3K9me2; (F) H3K9me3 and HP1α/ MacroH2A2 on human embryo cryosections. Scale bars, 20 μm.

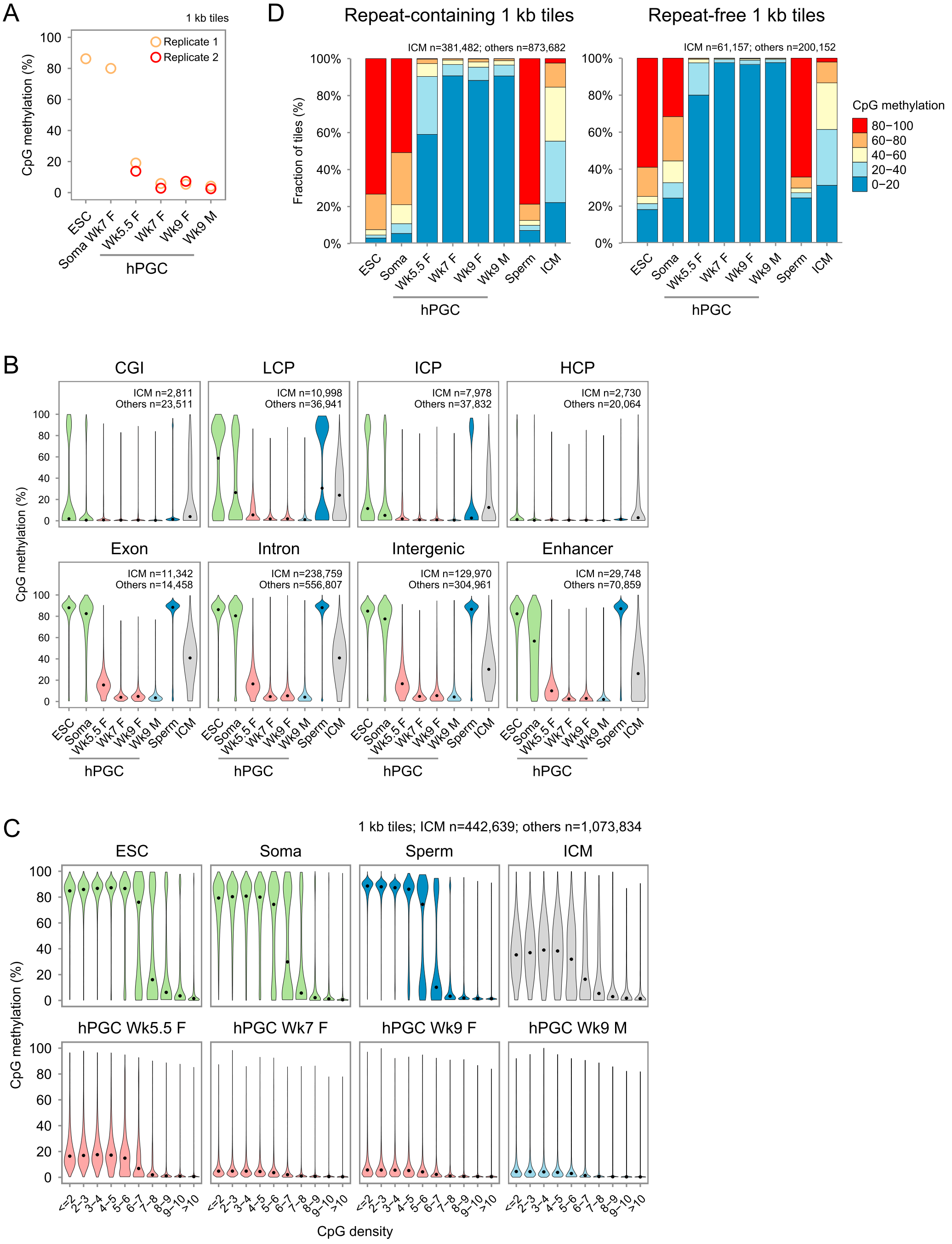

Figure S4. Comprehensive DNA Demethylation Revealed by Base-Resolution BS-Seq, Related to Figure 4

(A) Median CpG methylation levels of tiles in individual biological replicates of Wk5.5-Wk9 female and male hPGCs, ESC and soma. For soma and hPGCs, each replicate refers to cells collected from one individual embryo. Uncommon tiles with a minimum of 5 CpGs and at least 20% of the total CpGs covered by at least 5 reads in each sample are used.

(B) Distribution of CpG methylation levels in CpG islands (CGI), low-, intermediate-, and high-CpG density promoters (LCP, ICP and HCP respectively), exons, introns, intergenic regions and active enhancers of selected somatic tissues of NIH Roadmap Epigenomics Project.

(C) Distribution of methylation levels in tiles with various CpG densities. Note that tiles of Wk7-Wk9 hPGCs are globally demethylated regardless of CpG density.

(D) Distribution of methylation levels of repeat-containing and repeat-free 1 kb tiles. Note that a larger proportion of repeat-containing tiles retains partial methylation compared to repeat-free tiles.

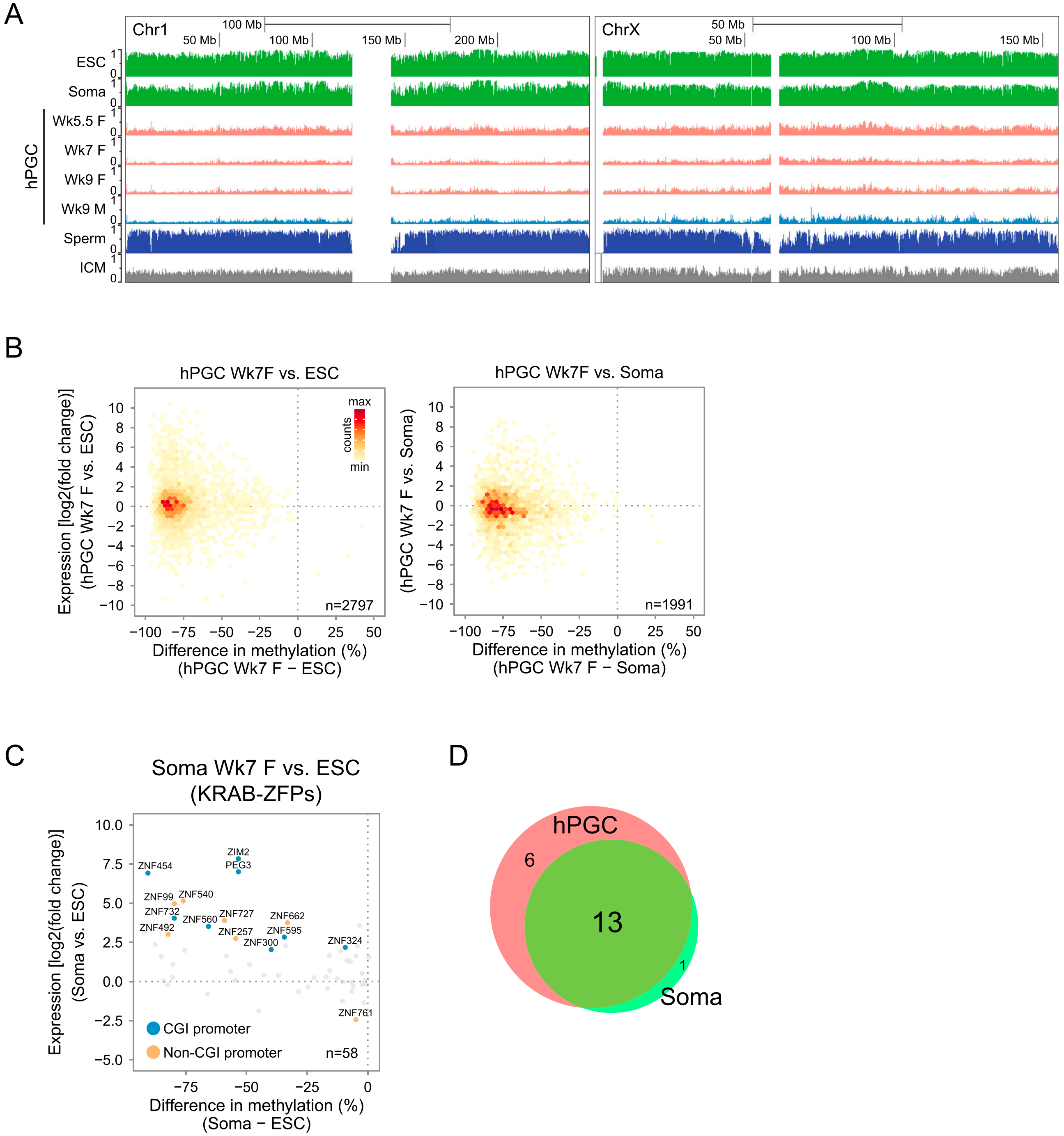

Figure S5. X Chromosome Demethylation and Regulation of Gene Expression by Promoter Methylation, Related to Figure 5

(A) UCSC genome browser screenshots of CpG methylation levels over whole chromosome 1 (left panel) and chromosome X (right panel)

(B) Hexagonal bin plots showing promoter demethylation in hPGCs is globally uncoupled with gene expression. Differential gene expression are plotted against difference in promoter methylation between Wk7 female hPGCs and ESC (left panel) or gonadal somatic cells (right panel). Color intensity indicates genes counts within each bin.

(C) Scatter plot of differential gene expression and difference in promoter methylation of KRAB-ZFPs between soma and ESCs. Interpro KRAB-ZFPs with > 20% promoter methylation and $\log_2$(read counts)>3 in either samples are shown. Colored points represent differentially expressed genes ($\log_2$(fold change)>2 and $p < 0.05$).

(D) Venn diagram showing overlap of upregulated KRAB-ZFPs in Wk7 F hPGCs or soma (refer to Figures 5G and S5C) against ESCs.

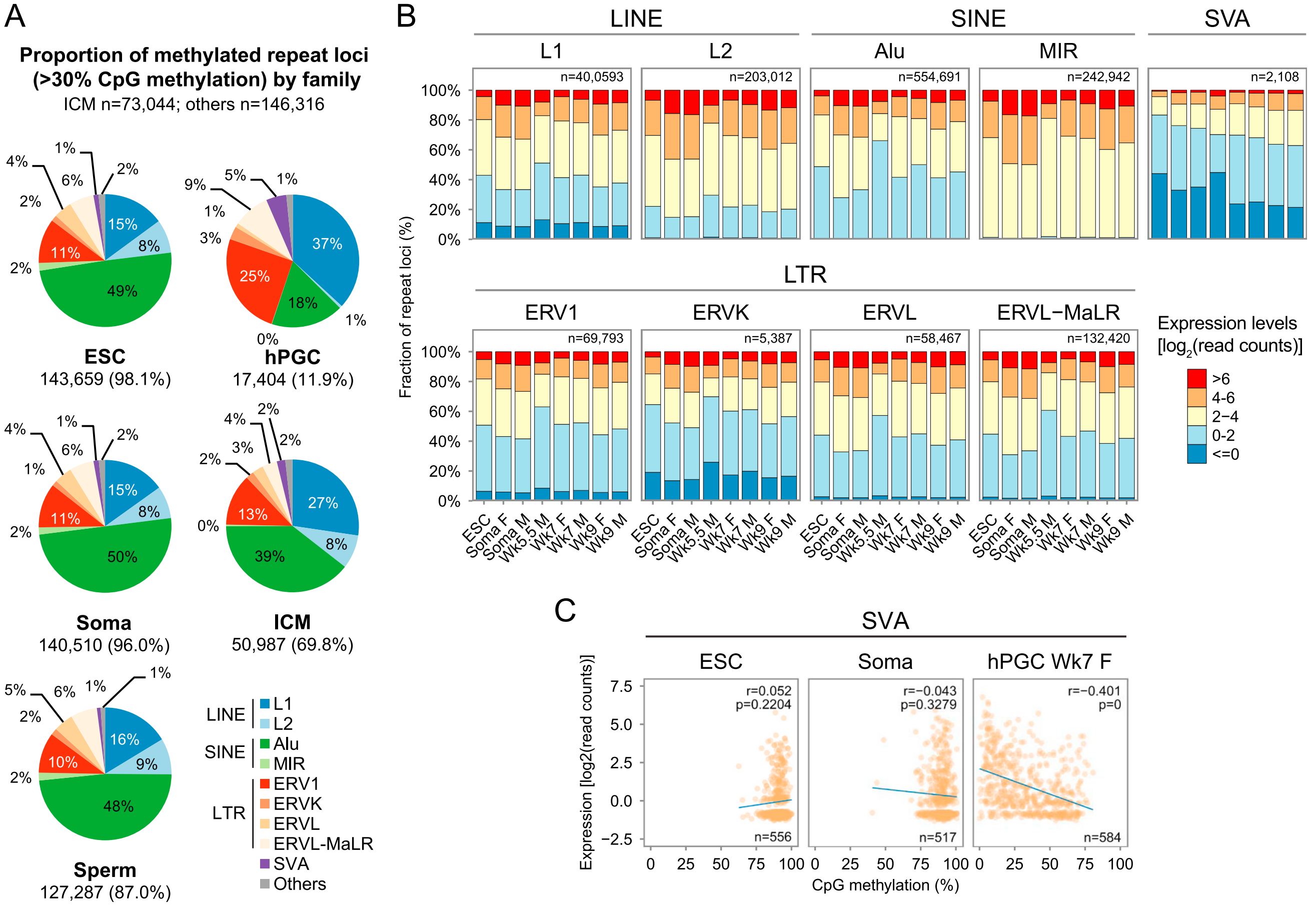

Figure S6. Demethylation and Transcriptional Dynamics of Retrotransposons, Related to Figure 6

(A) Pie charts showing distribution of major repeat subfamilies that retain CpG methylation (> 30%) in the indicated samples. For hPGCs, only repeat loci that are commonly methylated in week 7-9 are considered. Value under each pie chart indicates the number and percentage of repeats that are methylated. Note that methylated L1, ERV1 and SVA repeats are overrepresented in hPGCs.

(B) Distribution of RNA expression levels of major repeat families. Note that no global upregulation of repeats is observed in hPGCs upon genome-wide demethylation, except for SVA which showed a larger fraction of expressed loci at Wk7 and Wk9.

(C) Correlation of SVA subfamilies expression and methylation. “r” represents Pearson correlation coefficient. Note that moderate negative correlation of methylation and expression is observed in week 7 female hPGC. Linear regression is illustrated as blue line.

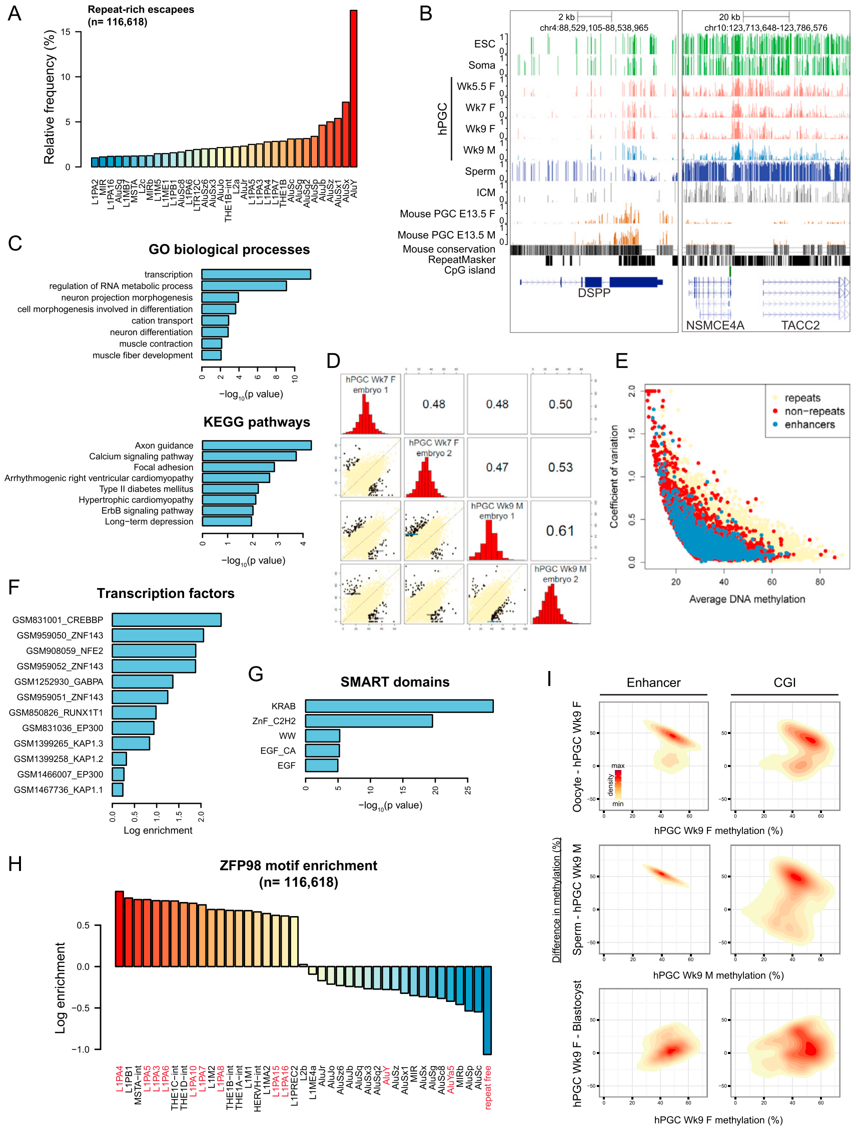

Figure S7. DNA Demethylation Escapees Are Candidates of Epigenetic Inheritance, Related to Figure 7

(A) Distribution of retrotransposon subfamilies in repeat-rich escapees. Note that there is a bias toward the most abundant and active subfamilies, including AluY and L1PAs.

(B) UCSC genome browser screenshots of two representative escapees in hPGCs. A region with retained DNA methylation of *DSPP* is conserved between human and mouse and intersects with low complexity simple repeats. *TACC2* has an escapee region across its promoter, which is not conserved in the mouse.

(C) DAVID enrichment analysis of GO biological processes and KEGG pathways of 2,092 protein-coding genes with repeat-poor escapees in their gene bodies. Top-enriched terms are shown for each category, redundant GO terms were removed.

(D) Scatter plot of inter-individual DNA methylation variation and disease association of repeat-poor escapees. Shown are 2,892 (out of 7,071) high-confidence repeat-poor escapees that have at least 40% DNA methylation in hPGCs of one of the individuals, at least 10 CpGs and at least 20% of the total CpGs in the region covered by 5X in all four individuals. Pearson correlation coefficients of escapee methylation levels between individuals are indicated.

(E) Coefficient of variation of methylation levels for repeat-rich, repeat-poor and enhancers-associated escapees, which have at least 40% DNA methylation in one of the individuals, at least 10 CpGs and at least 20% of the total CpGs in the region covered by 5X in all four individuals.

(F) Transcription factor binding sites enrichment of repeat-poor escapees. NCBI GEO accession number of each dataset are shown.

(G) DAVID enrichment analysis of SMART protein domains of 2,092 protein-coding genes with repeat-poor escapees in their gene bodies.

(H) Enrichment of ZFP98 motif among repeat-poor escapees and methylated retrotransposons (with > 700 copies).

(I) DNA methylation dynamics in gametes, blastocyst and hPGCs of repeat-poor escapees that intersect with annotated enhancers and CGIs. Methylation levels of CGI and enhancer escapees were in general amplified in gametes, especially for enhancer regions, which were fully re-methylated in sperm. However, these regions showed very similar partial methylation levels in blastocyst following resetting of DNA methylation in early embryos.
